# Phosphoinositide 3-Kinase p110 Delta Differentially Restrains and Directs Naïve Versus Effector CD8^+^ T Cell Transcriptional Programs

**DOI:** 10.3389/fimmu.2021.691997

**Published:** 2021-06-18

**Authors:** Laura Spinelli, Julia M. Marchingo, Aneela Nomura, Marcos P. Damasio, Doreen A. Cantrell

**Affiliations:** Division of Cell Signalling and Immunology, School of Life Sciences, University of Dundee, Dundee, United Kingdom

**Keywords:** PI3K, p110δ, CD8^+^ T cells, TCR signalling, transcriptomics, cytokines, chemokines

## Abstract

Phosphoinositide 3-kinase p110 delta (PI3K p110δ) is pivotal for CD8^+^ T cell immune responses. The current study explores PI3K p110δ induction and repression of antigen receptor and cytokine regulated programs to inform how PI3K p110δ directs CD8^+^ T cell fate. The studies force a revision of the concept that PI3K p110δ controls metabolic pathways in T cells and reveal major differences in PI3K p110δ regulated transcriptional programs between naïve and effector cytotoxic T cells (CTL). These differences include differential control of the expression of cytolytic effector molecules and costimulatory receptors. Key insights from the work include that PI3K p110δ signalling pathways repress expression of the critical inhibitory receptors CTLA4 and SLAMF6 in CTL. Moreover, in both naïve and effector T cells the dominant role for PI3K p110δ is to restrain the production of the chemokines that orchestrate communication between adaptive and innate immune cells. The study provides a comprehensive resource for understanding how PI3K p110δ uses multiple processes mediated by Protein Kinase B/AKT, FOXO1 dependent and independent mechanisms and mitogen-activated protein kinases (MAPK) to direct CD8^+^ T cell fate.

## Introduction

CD8^+^ cytotoxic T cells are critical cells for mammalian adaptive immune responses that clear pathogen infected cells. The proliferation and differentiation of CD8^+^ T cells into cytolytic effector cells (CTL) is directed by an integrated network of signals driven by the T cell antigen receptor (TCR) and modulated by cytokines and cytokine receptors and costimulatory and inhibitory receptors (1, 2). One rapid consequence of TCR engagement in CD8^+^ T cells is the production of phosphatidylinositol 3,4,5-trisphosphate (PtdIns(3,4,5)*P_3_*), the product of phosphoinositide 3-kinase (PI3K). CD8^+^ T cells thus rapidly accumulate PtdIns(3,4,5)*P_3_* following TCR engagement (3–5) and sustain high levels of PtdIns(3,4,5)*P_3_* as they differentiate to effector CTL (6, 7). Cellular levels of PtdIns(3,4,5)*P_3_* in T cells are controlled by a PI3K complex that contains the p110δ catalytic subunit (PI3K p110δ) (4, 8, 9). The importance of PI3K p110δ is emphasised by the discovery that both loss and gain of function mutations of PI3K p110δ cause primary immunodeficiency and autoimmunity in humans and mice (10–13). Effective PI3K p110δ inhibitors, used clinically to treat immune dysfunction and cancer, are available (14–17).

The importance of PI3K p110δ for T cells makes it important to understand the molecular details of how it controls T cell function. In this context, PtdIns(3,4,5)*P_3_* controls the activity of the serine/threonine kinase PKB/AKT which phosphorylates the transcription factor FOXO1 causing its exclusion from the nucleus and terminating FOXO1 controlled transcription. In naïve T cells, FOXO1 controls expression of transcription factors and cytokine receptors that maintain T cell quiescence (18). FOXO1 also controls expression of the adhesion molecules and chemokine receptors that instruct T cell trafficking to secondary lymphoid tissue  (19–21). In pathogen activated and effector CTL, PI3K p110δ signalling keeps FOXO1 cytosolic and inactive (22) to maintain CD8^+^ T cell effector function (23). The loss of PKB/AKT activity causes nuclear relocation of FOXO1 resulting in the induction of the transcriptional program required for memory T cells (18, 23). FOXO1 is thus essential for memory CD8^+^ T cell formation and maintenance (24–27) and may control T cell exhaustion during chronic viral infections by to controlling the expression of the inhibitory receptor PD-1 and regulating T cell survival (28).

FOXO1 studies have given important insights about how PI3K p110δ controls CD8^+^ T cells but it is pertinent that PI3K p110δ signalling pathways go beyond the control of FOXO1. There are thus multiple substrates for PKB/AKT and PI3K p110δ also modulates the activity of mitogen-activated protein (MAP) kinases (29, 30). Furthermore, there has never been an unbiased analysis or direct comparison of how PI3K p110δ shapes transcriptomes and proteomes in naïve versus effector CD8^+^ T cells. In this respect, signal transduction outcomes can be shaped by the epigenetic and metabolic remodelling that accompanies T cell differentiation. Hence in naïve T cells the role of the TCR is to initiate expression of biosynthetic and transcriptional programs that drive T cell clonal expansion and differentiation. In effector CD8^+^ T cells these programs are controlled by pro-inflammatory cytokines and the role of the TCR is to trigger execution of effector function (e.g. the production of cytokines and degranulation of cytolytic effector molecules).

Accordingly, the current objective was to use RNA sequencing, proteomic and secretome analysis to define the contribution of PI3K p110δ to antigen receptor regulated programs in naïve and effector CD8^+^ T cells. For mechanistic insights, we examined PI3K p110δ controlled responses in wild type (WT) and FOXO1 null effector CTL to understand how much of PI3K signalling is directed by FOXO1. We also assessed the importance of PKB/AKT and the MAP kinases as mediators of PI3K p110δ responses. This work provides a comprehensive understanding of the targets for PI3K p110δ signals in antigen receptor activated naïve and effector CD8^+^ T cells and creates the information framework necessary to predict the implications of the therapeutic manipulation of PI3K p110δ for CD8^+^ T cells.

## Materials and Methods

### Mice

For naïve and 24 h activated CD8^+^ T cells ± PI3K p110δ inhibitor, unstimulated and 4 h TCR activated CTL ± PI3K p110δ inhibitor transcriptomes, P14 transgenic mice (31) were used (female mice, 9–13 weeks old).

FOXO1-GFP^fl/fl^ mice were generated at Taconic. To allow conditional deletion of FOXO1, exon 2, which includes the EGFP sequence, was flanked by LoxP sites (32). Then to allow the deletion of FOXO1-GFP in effector T cells, FOXO1-GFP^fl/fl^ mice were crossed to mice expressing the Cre recombinase under the control of the granzyme B promoter sequence [**Supplementary Figure 5B** and (33, 34)] generating FOXO1-GFP^fl/fl^ GzmB cre mice.

Female FOXO1-GFP^fl/fl^ GzmB cre+ mice and control GzmB cre+ mice aged between 11 and 27 weeks were used for RNA sequencing of CTL cultured for 24 h in the presence or absence of inhibitor. In some experiments FOXO1-GFP^fl/fl^ GzmB cre− mice were used as controls. Experimental mice were age and sex matched. Mice were bred and maintained in the Biological Resource Unit at the University of Dundee using procedures that were approved by the University Ethical Review Committee and under the authorisation of the UK Home Office Animals (Scientific Procedures) Act 1986.

### Cell Cultures

All cells were activated and cultured in complete RPMI 1640 media containing glutamine (Thermo Fisher Scientific/Gibco), supplemented with 10% FBS (Gibco), penicillin/streptomycin (Gibco) and 50 µM ß-mercaptoethanol (Sigma). All cells were cultured at 37°C with 5% CO_2_.

–For *ex vivo* naïve CD8^+^ T cells, 24 h TCR activated and 24 h TCR activated in the presence of the PI3K p110δ inhibitor IC87114 (10 µM synthesized in-house), three mice were used per biological replicate. Cells from the three mice were pooled and then equally divided into the three conditions. Three biological replicates were generated per condition. Single cell suspensions were generated by mashing lymph nodes in RPMI media and filtered through a 70 µm cell strainer. For 24 h TCR activated CD8^+^ T cells, they were suspended in complete RPMI media with 100 ng/ml antigenic peptide [glycoprotein amino acids 33–41 (gp33–41)], 20 ng/ml interleukin-2 (IL-2; Proleukin, Novartis) and 2 ng/ml interleukin-12 (IL-12; PeproTech). Where indicated, cells were treated with 10 µM IC87114 inhibitor. DMSO was used as vehicle control. After 24 h, cells were harvested by centrifugation and prepared for cell sorting to isolate pure CD8^+^ T cell populations.

–To generate CTL from P14 transgenic mice, lymph nodes or spleens from three mice were used to generate three biological replicates. Lymph nodes were homogenised as described above. Spleens were mashed in red blood cell lysis buffer (150 mM NH_4_Cl, 10 mM KHCO_3_, 110 µM Na_2_EDTA pH 7.8), filtered through a 70 µm cell strainer and then suspended in complete RPMI media supplemented with 100 ng/ml gp33–41 peptide, 20 ng/ml IL-2 and 2 ng/ml IL-12 in 10 ml complete RPMI. Cells were activated for 48 h, then washed out and cultured for a further five days in complete RPMI media supplemented with 20 ng/ml IL-2. Cells were maintained at a cell density of 0.3 × 10^6^ live cells/ml.

For transcriptomic and proteomic studies and western blotting of TCR stimulated CTL: on day seven CTL were plated at a cell density of 1 × 10^6^ live cells/ml in complete RPMI media supplemented with 20 ng/ml IL-2 and left to rest at 37°C with 5% CO_2_ for 1 h. CTL were then stimulated by adding 100 ng/ml gp33–41 peptide directly to the supplemented media for 4 h in the presence or absence of IC87114 inhibitor (10 µM), or MEK inhibitor PD184252 (2 µM, Division of Signal Transduction Therapy (DSTT) Dundee). DMSO was used as vehicle control. Cells were harvested, washed twice with HBSS (Thermo Fisher Scientific/Gibco), snap frozen in liquid nitrogen and stored at −80°C until further processing.

For secretomic studies of TCR stimulated CTL: on day seven CTL were plated at a density of 1 × 10^6^ live cells/ml in serum free RPMI media (phenol red free, Thermo Fisher Scientific Gibco) supplemented with 50 µM ß-mercaptoethanol (Sigma), Insulin-Transferrin-Selenium 100× (ITS, Gibco) and 20 ng/ml IL-2 and left to rest at 37°C with 5% CO_2_ for 1 h. CTL were then stimulated as described above for 4 h in the presence or absence of 10 µM IC87114 inhibitor or 2 µM PD184252 inhibitor or 1 µM AKT inhibitor 1/2 (Merck chemicals). DMSO was used as vehicle control. Supernatants were then collected, passed through a 0.22 μm filter, snap frozen in liquid nitrogen and stored at −80°C until further processing.

For ELISA: CTL were plated at a density of 1 × 10^6^ live cells/ml in complete RPMI media supplemented with 20 ng/ml IL-2 and left to rest at 37°C with 5% CO_2_ for 1 h prior stimulation with 100 ng/ml gp33–41 peptide added to the supplemented media for 4 h in the presence or absence of 10 µM IC87114 inhibitor or 2 µM PD184252 inhibitor or 1 µM AKT inhibitor 1/2. DMSO was used as vehicle control. Supernatants were collected, snap frozen in liquid nitrogen and stored at −80°C until further processing.

–To generate CTL from FOXO1-GFP^fl/fl^ GzmB cre+ mice (FOXO1-GFP KO) and control GzmB cre+ mice (WT), lymph nodes or spleens were used and single cell suspensions were prepared as described above. Cells were activated with 0.5 µg/ml anti-mouse CD3 (clone 145-2C11 Biolegend), 0.5 µg/ml anti-mouse CD28 (clone 37.51 eBioscience), in 10 ml complete RPMI media supplemented with 20 ng/ml IL-2 and 2 ng/ml IL-12. Cells were activated for 48 h, then washed out and cultured for a further five days in complete RPMI media supplemented with 20 ng/ml IL-2 and 2 ng/ml IL-12.

For transcriptomic studies, on day six cells were plated at a density of 0.5 × 10^6^ live cells/ml in complete RPMI media supplemented with 20 ng/ml IL-2 and 2 ng/ml IL-12 and with or without 10 µM IC87114 for 24 h. DMSO was used as vehicle control. On day seven, media, containing all as above, was refreshed 2 h before cells were harvested and prepared for cell sorting.

–For FOXO1-GFP nuclear localisation: CTL from FOXO1-GFP^fl/fl^ GzmB cre− mice (expressing FOXO1-GFP tagged protein) were generated. Cells from WT mice (expressing untagged FOXO1 protein) were used as negative control for GFP expression. Spleens were used and single cell suspensions were prepared as described above. Cells were activated with 0.5 µg/ml anti-mouse CD3, 0.5 µg/ml anti-mouse CD28, in 10 ml complete RPMI media supplemented with 20 ng/ml IL-2 and 2 ng/ml IL-12. Cells were activated for 48 h, then washed out and cultured in complete RPMI media supplemented with 20 ng/ml IL-2. On days six or seven, cells were plated at a density of 1 × 10^6^ live cells/ml in the presence or absence of IC87114 inhibitor for 1 h (10 µM). DMSO was used as vehicle control.

### Cell Sorting

Cell sorting was performed on a Sony SH800S cell sorter (Sony Biotechnology). Staining was performed in PBS supplemented with 1% FBS. Sorting and cells collection for *ex vivo* naïve CD8^+^ T cells and 24 h TCR activated CD8^+^ T cell with or without inhibitor was performed in RPMI 1640 containing glutamine, supplemented with 1% FBS. Sorting and cell collection for FOXO1-GFP KO and WT CTL was performed in RPMI 1640 containing glutamine, supplemented with 10% FBS.

Fluorophore-conjugated antibodies were obtained from BD Biosciences, eBioscience or Biolegend.

–*Ex vivo* naïve CD8^+^ T cells were sorted as DAPI^-^TCRß^+^CD8^+^CD62L^hi^CD44^lo^. 1 µg Fc block (BD Pharmingen) per million cells was used to block Fc receptors. Cells were then stained with the fluorophore-conjugated antibodies TCRß-PerCPCy5.5 (H57-587), CD4-PECy7 (RM4-5), CD8-PE (53-6.7), CD44-APC (IM7), CD62L-FITC (MEL-14) and DAPI in PBS 1% FBS.
–24 h TCR activated CD8^+^ T cells with or without inhibitor were stained with the fluorophore-conjugated antibody CD8-APC and DAPI and sorted as DAPI^−^CD8^+^.
–CTL FOXO1-GFP KO from FOXO1-GFP^fl/fl^ GzmB cre+ mice were stained with the fluorophore-conjugated antibody CD8-APC and DAPI and sorted as DAPI^−^CD8^+^GFP^−^.
–CTL WT from GzmB cre+ mice were stained with the fluorophore-conjugated antibody CD8-APC and DAPI and sorted as DAPI^−^CD8^+^.
All sorted cells were then washed twice with HBSS before being snap frozen in liquid nitrogen and stored at −80°C until further processing.

### Forward/Side Scatter

Forward and side scatter profiles for sorted WT CTL treated or untreated for 24 h with the PI3K p110δ inhibitor were acquired on a FACSVerse using FACSuite software and data were analysed using FlowJo software version 10.6.1 (Treestar).

### Flow Cytometry Analysis of Nuclear Extract for FOXO1-GFP Localisation

Harvested cells were washed in PBS supplemented with 1% FBS and then either not lysed or lysed in 300 µl ice-cold nuclear extraction buffer (3.8 mM trisodium citrate, 9.6 mM NaCl, 0.05% NP-40). Total cells (used as control to show that PI3K p110δ inhibition increases FOXO1 nuclear localisation but not the total level of FOXO1 protein) and the nuclei of lysed cells were then fixed immediately with 300 µl ice-cold IC fixation buffer (eBioscience) for 15 min at 4°C. After washing, nuclei were stained with DAPI. Samples were acquired on Novocyte (Acea Biosciences Inc.) using Novo Express software. Data analysis was performed using FlowJo software version 10.6.1 (Treestar).

### ELISA

ELISA kits for GZMB, IFNγ and TNFα (Invitrogen) were used according to the manufacturer’s instructions to determine the effects of 4 h TCR stimulation and PI3K p110δ, MEK or AKT inhibitor treatments on the secretion of these proteins. Experiments were performed in biological triplicate. Statistical analysis was performed with Prism 7 for Mac OS X using unpaired, unequal variance t-test with Welch’s correction. The *P* values are considered as follow: *P <0.05, **P <0.01, ***P <0.001, ****P <0.0001, ‘ns’ not statistically significant.

### Western Blotting

Cell pellets were lysed in RIPA buffer (100 mM HEPES, pH 7.4, 150 mM NaCl, 1% NP40, 0.1% SDS, 0.5% sodium deoxycholate, 10% glycerol, 1 mM EDTA, 1 mM EGTA, 1 mM TCEP, and protease and phosphatase inhibitors (Roche)). Cell lysates were sonicated and then centrifuged at 4°C at 13,000 rpm for 10 min.

LDS sample buffer (1×) (Life Technologies) and tris(2-carboxyethyl)phosphine (TCEP, 25mM) was added to the samples prior to incubation at 100°C for 10 min. Samples were loaded and separated by SDS-PAGE (NuPAGE precast gels, Life Technologies), and then transferred to nitrocellulose membranes (Whatman). Blots were probed with the following antibodies: AKT p-S473 (Cell Signalling and Technology, #4058), AKT p-T308 (Cell Signalling and Technology, #4056), AKT (Cell Signalling Technology, #9272), SMC1 (Bethyl Laboratories, A300-055A), p44/42 MAPK p-T202/Y204, Cell Signalling and Technology, #9106) and p44/42 MAPK (Cell Signalling and Technology, #9102). Horseradish peroxidase (HRP)-conjugated secondary antibodies were used (Thermo Scientific). Chemiluminescence was measured using an Odyssey Fc Imaging System (Licor). Image Studio Software (LI-COR) was used to quantify chemiluminescence detected using the Odyssey system. Total protein bands were normalised to the corresponding SMC1 loading control, and then phosphorylated protein bands were normalised to the total protein band and normalised to a control sample (+TCR 4 h). Statistical analysis was performed with Prism 7 for Mac OS X using unpaired, unequal variance t-test with Welch’s correction. The *P* values are considered as follow: *P <0.05, **P <0.01, ***P <0.001, ****P <0.0001, 'ns' not statistically significant.

### RNA Extraction for RNA-Sequencing (RNAseq)

Total RNA was extracted from frozen cell pellets of sorted naïve CD8^+^ T cells and TCR activated with or without inhibitor, CTL either not TCR stimulated or TCR stimulated for 4 h in the presence or absence of inhibitor, and WT or FOXO1-GFP KO CTL with or without 24 h inhibitor treatment using Qiagen RNAeasy mini kit (Qiagen) as per manufacturer’s instructions. Total RNA was quantified using a Nanodrop ND-1000 spectrophotometer. Three biological replicates were generated for each condition.

For naïve CD8^+^ T cells less than 1 µg of total RNA was supplied; for TCR activated with or without inhibitor cells and all CTL samples 1 µg of total RNA was supplied to the Finnish Functional Genomics Centre of Turku Biosciences whom prepared the libraries and performed the sequencing.

### RNA Libraries Preparation and HiSeq Sequencing Run Information

The quality of the total RNA samples was inspected using Agilent Bioanalyzer 2100 or Advanced Analytical Fragment Analyzer and the concentration of the samples was measured with Nanodrop ND-2000 (Thermo Scientific) or Qubit Fluorometric Quantitation (Life Technologies). Libraries preparation was started from 300 ng of total RNA using the Illumina TruSeq Stranded mRNA sample preparation kit (15031047) as per manufacturer’s instructions. Data presented here were run as three independent experiments as follows: one lane for naïve/TCR 24 h activated cells with or without inhibitor (naïve/TCR activated 24 h ± inhibitor) (this lane included also an unpublished dataset not part of this manuscript); one lane for CTL unstimulated or TCR 4 h stimulated with or without inhibitors (Unstim/TCR stimulated 4 h CTL ± inhibitors); and one lane for WT and FOXO1-GFP KO CTL with or without inhibitor for 24 h (WT/FOXO1-GFP KO CTL ± inhibitor 24 h).

The quality of the libraries was confirmed with Advanced Analytical Fragment Analyzer and the concentrations of the libraries were quantified with Qubit Fluorometric Quantitation (Life Technologies). Libraries were sequenced using Illumina HiSeq 3000 (naïve/TCR activated ± inhibitor) or Illumina HiSeq 2500 (TCR stimulated CTL ± inhibitors) or Illumina HiSeq 3000 (WT/FOXO1-GFP KO CTL ± inhibitor) instruments using paired-end sequencing chemistry and 75 bp (naïve/TCR activated ± inhibitor) or 100 bp (TCR stimulated CTL ± inhibitors) or 75 bp (WT/FOXO1-GFP KO CTL ± inhibitor) read length. The typical yields are 120–150 × 10^6^ paired-end reads per lane on Illumina HiSeq 2500 and 260–310 × 10^6^ paired-end reads per lane on Illumina HiSeq 3000.

### RNAseq Data Processing

Naïve/TCR activated ± inhibitor and TCR stimulated CTL ± inhibitors: raw reads were first aligned against the *Mus musculus* mm10 reference genome using the STAR aligner (34) (versions 2.5.2b or 2.4.2a) and then associated with known genes using the RefSeq annotation. The number of reads associated with each gene was counted using subreads package (versions 1.5.1 and 1.5.0). Genome (*Mus Musculus* mm10) and reference gene annotations (RefSeq based annotation) were downloaded from Illumina iGenomes web site.

WT/FOXO1-GFP KO CTL ± inhibitor: read quality was evaluated and confirmed using MultiQC (35). Raw reads were aligned to the GRCm38.p6 (Ensembl) build of the Mus musculus reference genome using the STAR aligner (34) (version 2.7.1a), with ‘outFilterType BySJout’ option to reduce the number of spurious junctions. The STAR quantMode GeneCounts option was used to map aligned reads to genes to generate a gene counts matrix, using the annotation file GRCm38.98 (Ensembl).

For all experiments, the noise from low count genes was reduced using the filterByExpr function. The resulting count matrix was normalised by the Trimmed Mean of M-values (TMM) method using the edgeR package (36, 37). EdgeR RPKM function was used on TMM-normalised counts to calculate FPKM values, with total exon length calculated using GenomicRanges (38) as the sum of the non-overlapping exonic regions for a gene.

Differential expression analysis was performed using Limma package (39). Reads were converted to log_2_ counts per million and precision weighted using the voom function. A linear model was fitted to each gene and empirical Bayes moderated t-statistics were applied. *P* values were adjusted to control the global false-discovery rate.

All scripts used to perform analysis are available from authors upon request.

### Statistics and Calculations RNAseq Data

Genes were defined as differentially expressed if they had an adj. *P* value <0.05 and a fold change >2 or <0.5 (Naïve/TCR activated ± inhibitor and TCR stimulated CTL +/- inhibitors); and a fold change >1.5 or <0.67 (WT/FOXO1-GFP KO ± inhibitor). *adj. *P <*0.05 and fold change >2 or <0.5 and fold change >1.5 or <0.67.

For the gene ontology (GO) Term enrichment analysis, GO terms enriched in the statistically significant changed gene were identified using the functional annotation tools within DAVID Bioinformatics Resources 6.8, NIAID/NIH (http://david.ncifcrf.gov). The top 5 GO terms describing “biological process” annotation were identified based on *P* value and terms represented by <3 genes in the changed gene list were excluded from the analysis.

Heatmaps were generated using the Morpheus tool from the Broad Institute (http://software.broadinstitute.org/morpheus). Heatmap in **Figure 2B** was arranged with IFNγ positioned at the top and with all mRNA ranked according to similarity using the nearest-neighbor analysis and Pearson correlation. Heatmap in **Figure 2D** was arranged with Aacs positioned at the top. The other heatmaps, with the exclusion of heatmaps in **Figures 6F, G**, were arranged with the indicated mRNA on the top and the others ranked according to similarity using the nearest-neighbor analysis and Pearson correlation. Heatmaps in **Figures 6F, G** were arranged following the order of **Figures 3G, H**.

Venn diagrams were generated using Venny 2.1 from BioinfoGP (http://bioinfogp.cnb.csic.es/tools/venny) (40).

### Proteomics Sample Preparation

Cell pellets were lysed in 0.5 ml urea lysis buffer (8 M urea, 100 mM Tris pH 8.0, protease (cOmplete Mini EDTA-free, Roche) and phosphatase inhibitors (PhosSTOP, Roche) and vigorously mixed for 30 min at room temperature. Samples were sonicated with a Branson digital sonicator and protein concentration was determined by BCA assay as per manufacturer’s instructions (Thermo Fisher Scientific). Lysates were reduced with 10 mM dithiothreitol (DTT) and incubated at 30°C for 30 min before being alkylated with 50 mM iodoacetamide (IAA) in the dark at room temperature for 45 min. Lysates were then diluted with tris buffer (100 mM tris pH 8.0, 1 mM CaCl_2_) to 4 M urea. LysC (Wako), reconstituted in tris buffer, was added to the samples at a 50:1 (protein:LysC) ratio and incubated at 30°C overnight. Samples were then transferred to low-bind 15 ml Falcon tubes (Eppendorf) and further diluted with tris buffer to 0.8 M urea before trypsin (Promega), reconstituted with tris buffer, was added at a ratio of 50:1 protein/trypsin and incubated at 30°C for 8 h. After digestion, samples were adjusted to 1% trifluoroacetic acid (TFA) prior desalting with C18 Sep-Pack cartridges (Waters). The cartridges were washed twice with 1 ml elution buffer (50% acetonitrile ACN/0.1%TFA) and equilibrated twice with 1 ml wash buffer (0.1% TFA). Acidified peptides were loaded twice and then washed three times with 1 ml washing buffer. Peptides were eluted into Eppendorf Protein LoBind tubes by 2 elutions of 600 μl elution buffer each and dried down in a SpeedVac (Genevac).

### Strong Anion Exchange Chromatography

Dried peptide samples were resuspended in 210 μl SAX sample buffer (10 mM sodium borate–20% (v/v) acetonitrile), pH was adjusted to pH 9.3 with 1 M NaOH if necessary, and then fractionated with an UltiMate 3000 HPLC (high-performance liquid chromatography) system equipped with an AS24 SAX column. For the separation, the buffers used were 10 mM sodium borate (pH 9.3) (SAX buffer A) and 10 mM sodium borate (pH 9.3), 500 mM NaCl (SAX buffer B). An exponential elution gradient starting with Buffer A was used to separate the peptides into 16 fractions, which were collected and desalted with Sep-Pack C18 96-well desalting plates (Waters). Desalted peptides were then dried down with a SpeedVac (Genevac). Dried fractions were then resuspended in 1% formic acid, and separated by nanoscale C18 reverse-phase chromatography (UltiMate 3000 RSLCnano System, Thermo Fisher Scientific) before being electrosprayed into the linear trap quadrupole–Orbitrap mass spectrometer (LTQ Orbitrap Velos Pro; Thermo Fisher Scientific) as described previously (22). The chromatography buffers used were as follows: HPLC buffer A (0.1% formic acid), HPLC buffer B (80% acetonitrile and 0.08% formic acid), and HPLC buffer C (0.1% formic acid). Peptides were then loaded onto an Acclaim PepMap100 nanoViper C18 trap column (100 μm inner diameter, 2 cm; Thermo Fisher Scientific) in HPLC buffer C with a constant flow of 5 μl/min. After trap enrichment, peptides were eluted onto an EASY-Spray PepMap RSLC nanoViper, C18, 2 μm, 100 Å column (75 μm, 50 cm; Thermo Fisher Scientific) using the following buffer gradient: 2% B (0 to 3 min), 2 to 40% B (3 to 128 min), 40 to 98% B (128 to 130 min), 98% B (130 to 150 min), 98 to 2% B (150 to 151 min), and equilibrated in 2% B (151 to 180 min) at a flow rate of 0.3 μl/min. The eluting peptide solution was automatically electrosprayed into the coupled linear trap quadrupole–Orbitrap mass spectrometer (LTQ Orbitrap Velos Pro; Thermo Fisher Scientific) using an EASY-Spray nanoelectrospray ion source at 50°C and a source voltage of 1.9 kV (Thermo Fisher Scientific). The mass spectrometer was operated in positive ion mode. Full-scan MS survey spectra (mass/charge ratio, 335 to 1,800) in profile mode were acquired in the Orbitrap with a resolution of 60,000. Data were collected using data-dependent acquisition (DDA): the 15 most intense peptide ions from the preview scan in the Orbitrap were fragmented by collision-induced dissociation (normalized collision energy, 35%; activation Q, 0.250; activation time, 10 ms) in the LTQ after the accumulation of 5,000 ions. Precursor ion charge state screening was enabled, and all unassigned charge states as well as singly charged species were rejected. The lock mass option was enabled for survey scans to improve mass accuracy. MS was performed by the Proteomics Facility, University of Dundee, UK.

### Processing and Analysis of Proteomic Data

The data were processed, searched and quantified with the MaxQuant software package (version 1.6.10.43). Proteins and peptides were identified using a hybrid database from databases in Uniprot release 2020 06 as described in Marchingo et al. (41). The following search parameters were used: protein N-terminal acetylation, methionine oxidation, glutamine to pyroglutamate, and glutamine and asparagine deamidation were selected as variable modifications; carbamidomethylation of cysteine residues was set as a fixed modification; Trypsin and LysC were selected as the proteolytic enzymes; up to two missed cleavages were permitted; protein and PSM False discovery rates was set to 0.01 and matching of peptides between runs was switched off. Perseus software package (version 1.6.6.0) was used for data filtering and protein copy number quantification. Proteins were quantified from unique (found only in a specific protein group) and razor (peptides assigned to a specific protein group without being unique to that group) peptides. The data set was filtered to remove proteins categorised as “contaminants”, “reverse” and “only identified by site”. Copy numbers were calculated using the proteomic ruler plugin as described in Wisniewski et al. (42). Copy numbers of histones in a diploid mouse cell get assigned to the summed peptide intensities of all histones present in a sample. The ratio between the histone peptide intensity and the summed peptide intensities of the other identified proteins is then used to estimate copy number per cell for all identified proteins in the data set.

### Statistics and Calculations Proteomic Data

Three biological replicates were generated. *P* values were calculated *via* a two-tailed, unequal-variance t-test on log_10_ transformed copy number per cell values in Microsoft Excel. *P* values <0.05 were considered as being statistically significant. Fold change >2 or <0.5 were considered as cut-off. Heatmaps were generated using the Morpheus tool from the Broad Institute (http://software.broadinstitute.org/morpheus). *P <0.05 and fold change >2 or <0.5.

### Secretomics Sample Preparation

For this experiment, 400 μl of supernatant was used for each sample and peptides were generated using the S-Trap mini column method (Protifi). Briefly, samples were mixed on a shaker at 1,000 rpm at room temperature for 5 min after addition of 10 mM TCEP and 50 mM TEAB (Triethylammonium bicarbonate) and then transferred to a ThermoMixer shaking at 500 rpm at 95°C for 5 min before being mixed again at 1,000 rpm at room temperature for 5 min. Samples were alkylated with 20 mM Iodoacetamide (IAA) in the dark at room temperature for 1 h and protein concentration was determined using the EZQ method (Thermo Fisher Scientific) according to the manufacturer’s instructions. Lysates were acidified by addition of 1.2% phosphoric acid and then mixed by vortexing. S-Trap binding buffer (90% methanol and 100 mM TEAB pH 7.1) was added at a 1:7 ratio of lysate to S-Trap binding buffer, loaded on S-Trap mini columns (Protifi) and then centrifuged at 6,500 rpm for 30 s. Bound protein was washed five times by addition of 400 μl of S-Trap binding buffer and centrifugation at 6,500 rpm for 30 s. Digestion buffer (125 μl) containing trypsin in a ratio protein 1:trypsin 20 was added into the top of the column and incubated at 47°C for 2 h without shaking. Peptides were then eluted by addition and centrifugation of 80 μl of digestion buffer, 80 μl of 0.2% aqueous formic acid and 80 μl of 50% aqueous acetonitrile containing 0.2% formic acid. Eluted peptides were dried down in a SpeedVac (Genevac) and then reconstituted in 5% formic acid by shaking at 1,000 rpm at 30°C for 1 h. Peptides were run on a Q Exactive Plus Hybrid Quadrupole-Orbitrap mass spectrometer (Thermo Fisher Scientific) as single shot in a data-dependent acquisition (DDA) mode (120 min). MS was performed by the Proteomics Facility, University of Dundee, UK. The data were processed, searched and quantified as described above with only trypsin selected as proteolytic enzyme. Raw intensity for each replicate was normalised to the corresponding control and expressed as % intensity relative to TCR. Statistical analysis was performed with Prism 7 for Mac OS X using unpaired, unequal variance t-test with Welch’s correction. The *P* values are considered as follow: *P <0.05, **P <0.01, ***P <0.001, ****P <0.0001.

## Results

### PI3K p110δ Control of Antigen Receptor Transcriptional Programs in Naïve CD8^+^ T Cells

**Figure 1:**
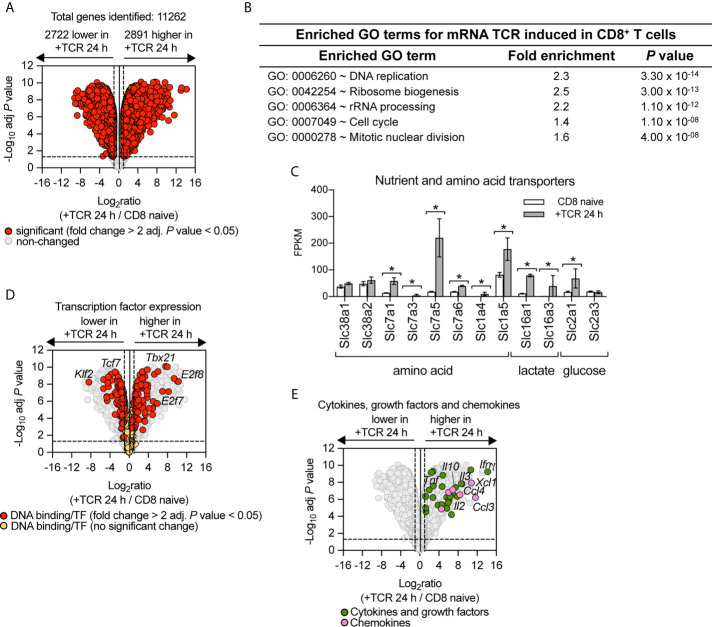
Remodelling of CD8^+^ T cell transcriptome in response to immune activation. RNA sequencing was used to characterise the transcriptome of sorted *ex vivo* naïve or 24 h gp33–41 peptide activated (TCR) P14 CD8^+^ T cells. **(A)** Volcano plot of mRNA expression of 24 h TCR activated versus naïve CD8^+^ T cells. mRNA significantly different between the two populations (fold change >2 or <0.5; adj. P values <0.05) shown in red. The horizontal dashed lines indicate an adj. *P* value of 0.05 shown as −log_10_ while vertical dashed lines indicate a fold change of 2 and 0.5 shown as log_2_. The vertical solid line indicates the mean of log_2_ ratio. **(B)** Enrichment analysis for biological processes for mRNA whose expression was induced upon TCR activation. The top 5 processes are presented. **(C)** Expression profile (FPKM) of amino acid, lactate and glucose transporters. FPKM shown as the mean of three biological replicates ± standard deviation. * adj. P <0.05 and fold change >2 or <0.5. **(D)** Volcano plot highlighting changes in transcription factor expression in response to 24 h TCR activation (GO:0003700 DNA binding transcription factor activity). Annotated mRNA significantly different between the two populations compared are shown in red (fold change >2 or <0.5; adj. *P* value <0.05). Transcription factors that showed no significant differences are shown in yellow. **(E)** Volcano plot highlighting mRNA expression of cytokines, growth factors and chemokines whose expression was induced in response to 24 h TCR activation (GO:0005125 Cytokine activity and GO:0008009 Chemokine activity). Annotated cytokines and growth factors (green) and annotated chemokines (pink) that were significantly upregulated upon TCR stimulation (fold change >2 or <0.5; adj. *P* value <0.05). Input data for highlighted mRNA in **(D, E)** are listed in **Supplementary Datasheet 1**.

TCR driven activation of naïve T cells is the key step in directing the differentiation of effector T cells. Therefore, to understand how PI3K p110δ controls CD8^+^ T cell differentiation we first assessed the contribution of PI3K p110δ signalling pathways to the transcriptional remodelling initiated by the TCR. The cell model used was P14 CD8^+^ T cells expressing a TCR specific for lymphocytic choriomeningitis virus (LCMV) glycoprotein peptide gp33–41. To define the PI3K p110δ transcriptional programs we used RNA sequencing to examine the transcriptome of naïve CD8^+^ T cells before and after 24 h of activation with the LCMV glycoprotein peptide gp33–41 in the presence or absence of the selective PI3K p110δ inhibitor IC87114 (PI3Kdi). In these experiments we quantified 11,262 mRNA and using a fold change cut off >2 fold and adj. *P* value <0.05 we saw that the abundance of 5,613 mRNA was modified by antigen receptor engagement (**Figure 1A**; **Supplementary Table 1** and **Supplementary Datasheet 1**). There are a number of mRNA (2,722), which represent around 24% of the total transcriptome measured, that are expressed in naïve T cells and downregulated upon immune activation including mRNA encoding transcription factors which maintain pluripotency and cell quiescence (e.g. *Klf2* and *Tcf7*), translational repressors such as *Pdcd4* and growth factor receptors such as the *Il7* and *Il6* receptors (**Supplementary Table 1**). There are also a large number of mRNA (2,891), around 26% of the total transcriptome measured, whose abundance increases following immune activation and this includes mRNA encoding proteins annotated in a GO term enrichment analysis as cell cycle machinery and ribosomal biogenesis (**Figure 1B** and **Supplementary Table 1**) but also includes nutrient transporters (**Figure 1C**), transcription factors (**Figure 1D**) and a wide array of cytokines, growth factors and chemokines (**Figure 1E**).

**Figure 2:**
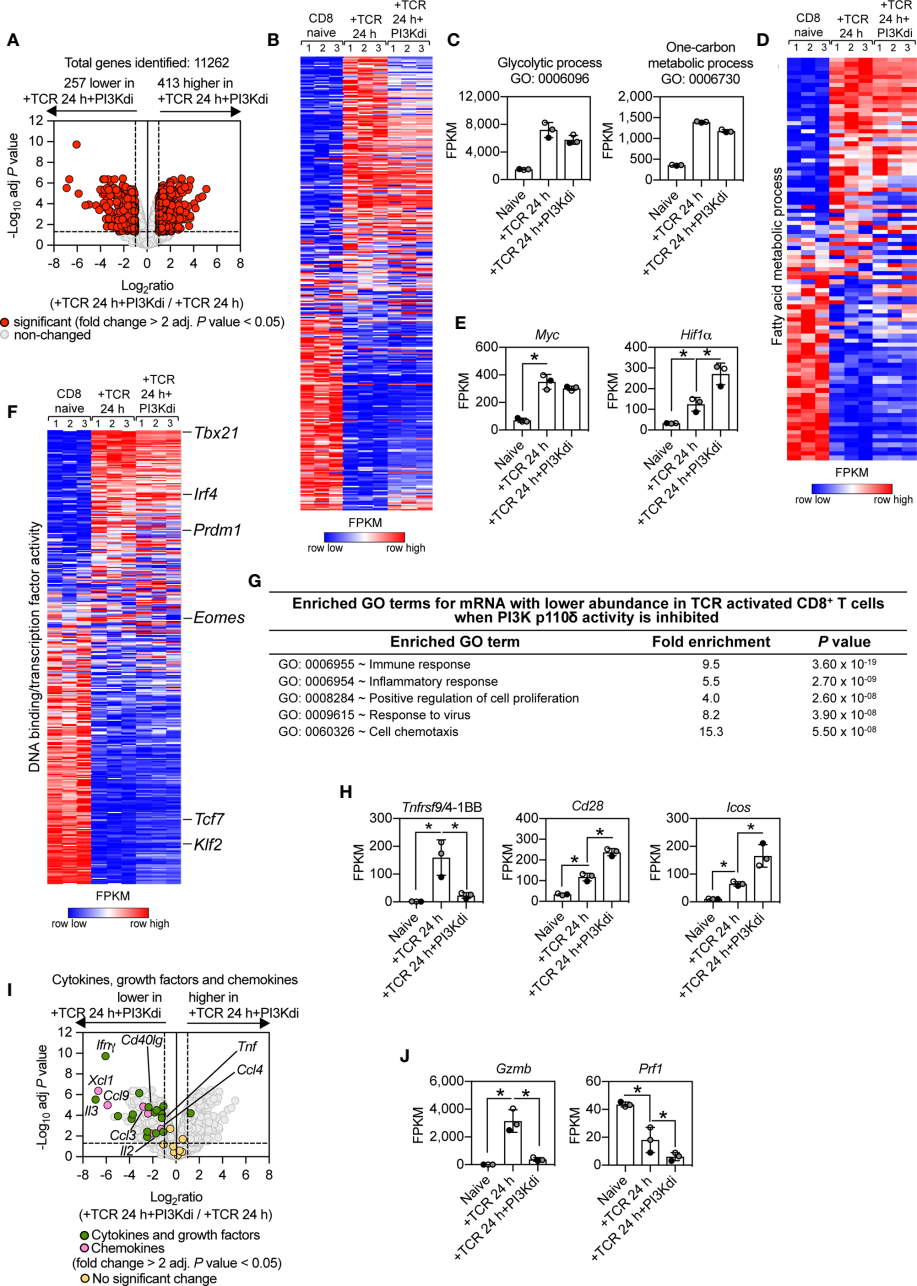
PI3K p110δ control of antigen receptor transcriptional programs in naïve CD8^+^ T cells. P14 CD8^+^ T cells were activated *in vitro* with the gp33–41 peptide in the presence or absence of the PI3K p110δ inhibitor IC87114 (PI3Kdi) for 24 h. **(A)** Impact of PI3K p110δ inhibition on mRNA expression. Volcano plot shows the ratio of PI3K p110δ treated versus untreated TCR activated CD8^+^ T cells. mRNA significantly different between the two populations (fold change >2 or <0.5; adj. *P* values <0.05) shown in red. The horizontal dashed lines indicate an adj. *P* value of 0.05 shown as −log_10_ while vertical dashed lines indicate a fold change of 2 and 0.5 shown as log_2_. The vertical solid line indicates the mean of log_2_ ratio. **(B)** Heatmap of CD8^+^ T cells naïve, TCR activated 24 h ± PI3K p110δ inhibitor (PI3Kdi) transcriptomes. Heatmap was arranged with *Ifnγ* positioned at the top and shows the relative mRNA abundance graded from low (blue) to high (red) per row. Input data for the heatmap are listed in **Supplementary Datasheet 1**. **(C)** Summed mRNA expression (FPKM) of genes annotated as glycolytic process (GO:0006096), and one-carbon metabolic process (GO:0006730). FPKM represented as the mean of three biological replicates ± standard deviation. Input data are listed in **Supplementary Datasheet 1**. **(D)** Heatmap of mRNA encoding enzymes that mediate fatty acid metabolism (Fatty acid metabolic process GO:0006631). The heatmap shows the relative mRNA abundance graded from low (blue) to high (red) per row. Input data for the heatmap are listed in **Supplementary Datasheet 1**. **(E, H, J)** mRNA expression levels (FPKM) of *Myc*, *Hif1α*, *Tnfrsf9*/4-1BB, *Cd28*, *Icos*, *Gzmb* and *Prf1*. FPKM shown as the mean of three biological replicates ± standard deviation. * adj. P <0.05 and fold change >2 or <0.5. **(F)** Heatmap of more than 300 mRNA annotated as DNA binding/transcription factor activity or added manually (GO:0003700). Heatmap was arranged with *Tbx21* mRNA positioned at the top and shows the relative mRNA abundance graded from low (blue) to high (red) per row. Input data for the heatmap are listed in **Supplementary Datasheet 1**. **(G)** Enrichment analysis for biological processes for mRNA whose expression was reduced in TCR activated CD8^+^ T cells by PI3K p110δ inhibition. The top 5 processes are presented. **(I)** Volcano plot highlighting the effect of PI3K p110δ inhibition on mRNA expression of cytokines, growth factors and chemokines induced in response to 24 h TCR activation (GO:0005125 Cytokine activity and GO:0008009 Chemokine activity). Shown in green are annotated cytokines and growth factors and shown in pink are annotated chemokines that were significantly regulated in response to PI3K p110δ inhibition (fold change >2 or <0.5; adj. P value <0.05). Cytokines, growth factors and chemokines that showed no significant differences are shown in yellow. Input data are listed in **Supplementary Datasheet 1**.

What are the consequences of inhibiting PI3K p110δ during TCR activation on the CD8^+^ T cell transcriptome? Overall, PI3K p110δ inhibition controlled the expression of 670 mRNA (>2 fold change threshold and an adj. *P* value of <0.05) in the TCR activated CD8^+^ T cells (**Figure 2A**), which represent 6% of the total 11,262 mRNA identified. There are 257 mRNA whose expression is repressed and more than 400 mRNA whose expression is increased by loss of PI3K p110δ activity (**Supplementary Table 2** and **Supplementary Datasheet 1**). The selectivity of the effects of PI3K p110δ inhibition is striking as shown by **Figure 2B** which uses nearest neighbor analysis and Pearson correlation to group and align the expression profile of 11,262 mRNA in naïve, TCR activated CD8^+^ T cells and CD8^+^ T cells activated *via* their TCR in the presence of the PI3K p110δ inhibitor. These data show that a large portion of the TCR regulated transcriptional programs is PI3K p110δ independent. Of note, PI3K p110δ does not mediate TCR induced changes in the abundance of transcripts encoding the metabolic machinery that mediate glycolysis, one carbon or fatty acid metabolism (**Figures 2C, D** and **Supplementary Figure 1A**). Moreover, TCR engagement switches on expression of the transcription factors *Myc* and *Hif1* alpha (*Hif1α*), which control expression of amino acid transporters and glucose transporters (41, 43, 44) respectively, even in the absence of PI3K p110δ activity (**Figure 2E**). Indeed expression of *Hif1α* mRNA was increased relative to controls in cells activated in the absence of PI3K p110δ activity. The selectivity of the effects of PI3K p110δ signalling is also highlighted by exploring the impact on the expression of mRNA encoding key transcription factors that control CD8^+^ T cell differentiation: over 300 transcription factors were identified using the annotation DNA binding/transcription factor activity (GO:0003700) and only 31 are controlled by PI3K p110δ (**Figure 2F** and **Supplementary Datasheet 1**). As examples, the ability of the TCR to downregulate expression of *Klf2* and *Tcf7* is not PI3K p110δ dependent. Nor does PI3K p110δ inhibition prevent TCR induction of *Tbx21*, *Blimp1* and *Irf4*: key drivers of CD8^+^ T cell differentiation (**Figure 2F** and **Supplementary Figure 2A**). Nevertheless, there are some key CD8^+^ T cell transcription factors whose expression is PI3K p110δ dependent including *Eomes* (**Figure 2F** and **Supplementary Figure 2A**). There are also increases in expression of mRNA encoding the transcriptional repressors BACH2 and ID2 in PI3K p110δ inhibitor treated cells (**Figure 2F** and **Supplementary Figure 2A**).

What are the PI3K p110δ controlled processes in T cells? Here GO term enrichment analysis reveals the dominant effect of PI3-kinase signalling pathways in TCR activated cells is to control expression of transcripts annotated as ‘immune response molecules’ (**Figure 2G**). For example, TCR triggering induces an increase in expression of mRNA encoding the important T cell costimulatory molecules 4-1BB, CD28 and ICOS: TCR mediated upregulation of mRNA encoding 4-1BB is dependent on PI3K p110δ; whereas *Icos* and *Cd28* mRNA is increased in PI3K p110δ inhibitor treated cells (**Figure 2H**).

Antigen activation also causes T cell to switch on expression of mRNA encoding a diverse array of cytokines, chemokines and cytotoxic effector molecules. These include Interleukin-2 (IL-2), Interferon gamma (IFN*γ*), Tumour necrosis factor (TNF), Interleukin-3 (IL-3), Interleukin-24 (IL-24), Lymphotoxin-alpha (LTα), Leukemia inhibitory factor (LIF), CD40 ligand (CD40lg), Chemokine (C-C motif) ligand 3 (CCL3), Chemokine (C-C motif) ligand 4 (CCL4), Chemokine (C motif) ligand 1 (XCL1). The induction of this subset of mRNA is all dependent on activation of PI3K p110δ (**Figure 2I**). The expression of mRNA encoding effector CD8^+^ T cell cytolytic molecules like granzymes and perforin also depends on PI3K p110δ activity in TCR activated CD8^+^ T cells (**Figure 2J**).

PI3K p110δ activation thus both positively and negatively controls the expression of T cell costimulatory molecules and is required for the production of multiple cytokines and effector molecules that allow CD8^+^ T cells to mediate adaptive immune responses. It is particularly striking that PI3K p110δ activity is required for the production of CCL3, CCL4 and XCL1 as these are the essential chemokines used by immune activated CD8^+^ T cells to direct their interactions with dendritic cells/monocytes and macrophages (45, 46).

### PI3K p110δ Control of Transcriptional Programs in Effector Cytotoxic CD8^+^ T Cells

**Figure 3:**
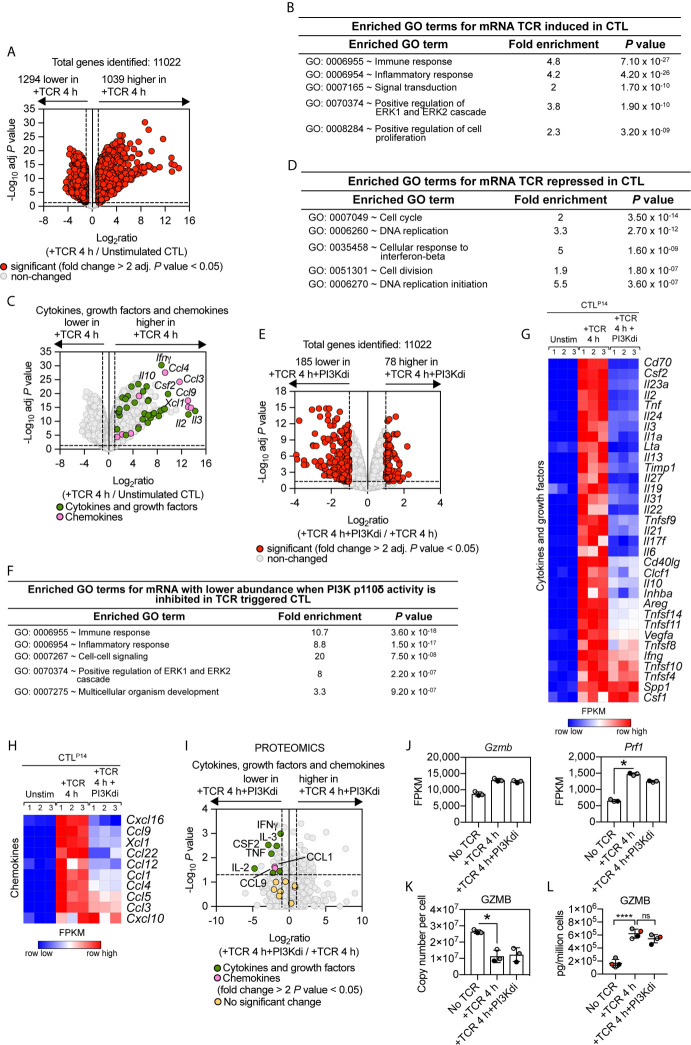
PI3K p110δ control of antigen receptor transcriptional programs in effector cytotoxic CD8^+^ T cells. P14 CTL were either left unstimulated or TCR retriggered with the LCMV peptide in the presence or absence of the PI3K p110δ inhibitor IC87114 (PI3Kdi) for 4 h and the transcriptome analysed by RNAseq. **(A)** Impact of TCR retriggering on mRNA expression in CTL. Volcano plot showing the ratio for 4 h TCR retriggered versus unstimulated CTL. mRNA significantly different between the two populations (fold change >2 or <0.5; adj. *P* values <0.05) shown in red. The horizontal dashed lines indicate an adj. *P* value of 0.05 shown as −log_10_ while vertical dashed lines indicate a fold change of 2 and 0.5 shown as log_2_. The vertical solid line indicates the mean of log_2_ ratio. **(B, D)** GO term enrichment analysis for biological processes for mRNA whose expression was up or downregulated by TCR retriggering. The top 5 processes are presented. **(C)** Volcano plot of cytokines, growth factors and chemokines mRNA whose expression was induced in CTL by 4 h TCR retriggering (GO:0005125 Cytokine activity and GO:0008009 Chemokine activity). Annotated cytokines and growth factors (green) and annotated chemokines (pink) that were significantly upregulated upon TCR retriggering (fold change >2 or <0.5; adj. *P* value <0.05). Input data are listed in **Supplementary Datasheet 2**. **(E)** Volcano plot showing the ratio of TCR stimulated CTL ± PI3K p110δ inhibitor (PI3Kdi). In red mRNA significantly different between the two populations (fold change >2 or <0.5; adj. *P* values <0.05). The horizontal dashed lines indicate an adj. *P* value of 0.05 shown as −log_10_ while vertical dashed lines indicate a fold change of 2 and 0.5 shown as log_2_. The vertical solid line indicates the mean of log_2_ ratio. **(F)** Enriched GO terms for mRNA whose expression was reduced in TCR retriggered CTL in the absence of PI3K p110δ activity. The top 5 biological processes are presented. **(G, H)** Heatmaps of mRNA encoding **(G)** cytokines, growth factors and **(H)** chemokines annotated as Cytokine activity (GO:0005125) and Chemokine activity (GO:0008009). The heatmaps show the relative mRNA abundance graded from low (blue) to high (red) per row. Input data for heatmaps are listed in **Supplementary Datasheet 2**. **(I)** The proteome of P14 CTL either left unstimulated or TCR retriggered for 4 h in the presence or absence of the PI3K p110δ inhibitor IC87114 (PI3Kdi) was analysed by mass spectrometry. Volcano plot shows fold change in protein copy number between TCR retriggered CTL lacking PI3K p110δ activity and TCR retriggered CTL, highlighting the effect of PI3K p110δ inhibition on the expression of TCR induced cytokine, growth factor and chemokine proteins. In green annotated cytokines and growth factors and in pink annotated chemokines that were significantly regulated in response to PI3K p110δ inhibition (fold change >2 or <0.5; *P* value <0.05). In yellow cytokines, growth factors and chemokines that showed no significant changes. Input data are listed in **Supplementary Datasheet 4**. **(J)** mRNA expression levels (FPKM) of *Gzmb* and *Prf1*. FPKM shown as the mean of three biological replicates ± standard deviation. *adj. P <0.05 and fold change >2 or <0.5. **(K)** Mean protein copy number per cell of granzyme B (GZMB) calculated using the proteomic ruler method as described in *Materials and Methods*. *P <0.05 and fold change >2 or <0.5. **(L)** Granzyme B levels in supernatants of P14 CTL unstimulated and TCR retriggered in the presence or absence of PI3K p110δ inhibitor (PI3Kdi) determined by ELISA. Data shown as the mean of four biological replicates ± standard deviation and statistical analysis calculated by unpaired, unequal variance t-test with Welch’s correction. The P values are considered asfollow: ****P < 0.0001 and ‘ns’ not statistically significant.

The TCR-PI3K p110δ signalosome is remodelled during T cell differentiation (47) but the contribution of PI3K p110δ to TCR triggered transcriptional programs in effector CD8^+^ T cells has not been assessed. To address this question, antigen-primed P14 TCR transgenic CD8^+^ T cells were clonally expanded in IL-2 to produce differentiated CTL. RNA sequencing then compared the transcriptome of CTL before and after retriggering of the TCR with the LCMV glycoprotein peptide gp33–41 in the presence or absence of the PI3K p110δ inhibitor IC87114. The data show that out of 11,022 mRNA, TCR triggering induces increased expression of 1,039 mRNA and downregulated expression of 1,294 mRNA (fold change cut off >2 and an adj. *P* value <0.05), representing just over 20% of the total transcriptome measured (**Figure 3A**; **Supplementary Table 3** and **Supplementary Datasheet 2**). The upregulated mRNA in TCR activated CTL encode cytokines, growth factors and chemokines highlighting how one key role of antigen receptor triggering in effector T cells is to produce molecules that communicate pathogen recognition to other cells of the immune system (**Figures 3B, C**). The downregulated mRNA in TCR activated CTL encode genes annotated in a GO term enrichment analysis as “cell cycle” (**Figure 3D**) that includes E2F transcription factors (*E2f1-E2f2-E2f7-E2f8*), cyclin A2 (*Ccna2*), cyclin B2 (*Ccnb2*) and cyclin D3 (*Ccnd3*) (**Supplementary Figures 3A, B**).

As in the naïve T cells, only a small fraction (less than 3%) of the TCR induced transcriptional programs is sensitive to inhibition of PI3K p110δ in CTL. Blocking PI3K p110δ activity decreased expression of approximately 185 mRNA and increased the abundance of 78 mRNA (**Figure 3E**, **Supplementary Table 4** and **Supplementary Datasheet 2**). Enrichment analysis of the downregulated mRNA revealed that the top 3 enriched GO terms for PI3K p110δ sensitive processes are biological processes involving cytokines and chemokines (**Figures 3F–H**). **Figure 3G** thus shows that PI3K p110δ activity is required for the production of mRNA encoding multiple cytokines including Granulocyte–macrophage colony-stimulating factor (GM-CSF/CSF2), IL-2, TNF, IL-3, IL-24, IL-10, Ltα, CD40lg. Notably, PI3K p110δ activity is also required for the TCR induced production of chemokine mRNA including CCL9 and XCL1 (**Figure 3H**). Moreover, the effects of PI3K p110δ inhibition on expression of mRNA encoding chemokines and cytokines were paralleled by changes in the production of cytokine and chemokine proteins as measured by high resolution mass spectrometry analysis of the proteomes and secretomes of unstimulated or P14 CTL activated with cognate peptide for 4 h in the presence or absence of the PI3K p110δ inhibitor (**Figure 3I** and **Supplementary Datasheet 4**).

In TCR activated naïve T cells, PI3K p110δ activity directed expression of the cytolytic effector molecules granzyme B and perforin (**Figure 2J**). mRNA and protein levels for these effector molecules are already high in CTL (**Figure 3J**) [**Supplementary Figure 4A** and (48)] However, neither granzyme nor perforin expression is PI3K p110δ regulated in CTL (**Figure 3J**). Here, an important consideration is that a primary function of TCR engagement in CTL is to induce degranulation of cytolytic effector molecules. TCR activated CTL thus lose intracellular granzyme B protein following TCR engagement (**Figure 3K**). This response is not prevented by PI3K p110δ inhibition, with the same amount of granzyme B content remaining in the cell in CTL after 4 h retriggering with or without inhibitor (**Figure 3K**). Analysis of granzyme B levels in cell supernatants also failed to see an impact of PI3K p110δ inhibition on granzyme B exocytosis (**Figure 3L**). PI3K p110δ thus does not control the TCR mediated production of cytolytic T cell granules or degranulation processes in CTL.

### The Impact of Sustained Loss of PI3K p110δ Activity on CTL Transcriptional Programs

**Figure 4:**
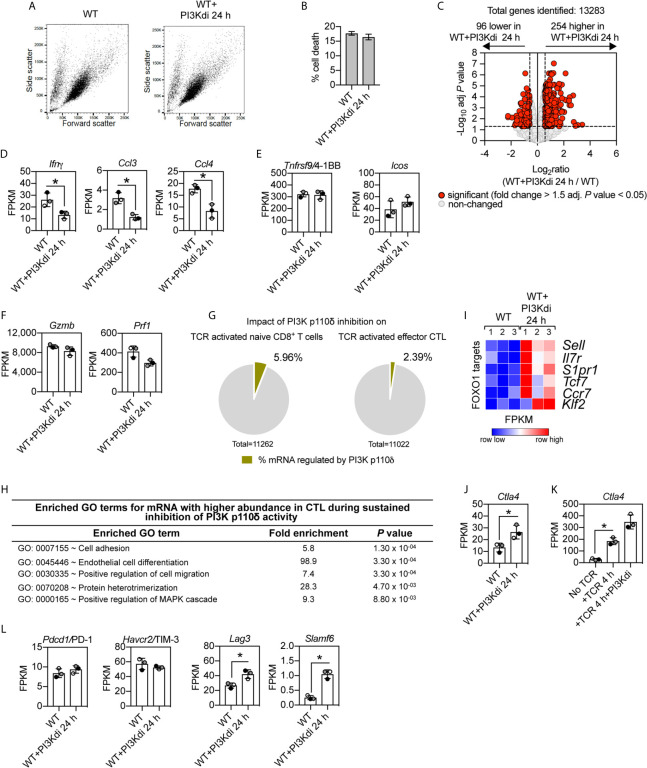
Impact of sustained PI3K p110δ inhibition on CTL transcriptional programs. WT CTL were maintained for 24 h in the presence or absence of the PI3K p110δ inhibitor IC87114 (PI3Kdi) and RNAseq was used to characterise their transcriptome. **(A)** Forward/side scatter flow cytometry analysis of IL-2/IL-12 maintained or 24 h inhibitor treated WT CTL. **(B)** Percentage of cell death at the end of the culture quantified by flow cytometry as proportion of cells DAPI positive. **(C)** Volcano plot showing the ratio for 24 h PI3K p110δ inhibitor (PI3Kdi) treated cells versus untreated WT CTL. mRNA significantly different between the two populations shown in red (fold change >1.5 or <0.67; adj. *P* values <0.05). The horizontal dashed lines indicate an adj. *P* value of 0.05 shown as −log_10_ while vertical dashed lines indicate a fold change of 1.5 and 0.67 shown as log_2_. The vertical solid line indicates the mean of log_2_ ratio. **(D–F)** Histograms showing mRNA expression levels (FPKM) for *Ifnγ*, *Ccl3*, *Ccl4*, *Tnfrsf9/*4-1BB, *Icos*, *Gzmb* and *Prf1*. FPKM shown as the mean of three biological replicates ± standard deviation. **(G)** Percentage of the impact of PI3K p110δ inhibition on the total transcriptome of TCR activated naïve (left) and effector CD8^+^ T cells (right). **(H)** GO term enrichment analysis for biological processes for mRNA whose expression was induced by sustained inhibition of PI3K p110δ activity. The top 5 processes are presented. **(I)** Heatmap of known FOXO1 targets in WT CTL cultured for 24 h in the presence or absence of PI3K p110δ inhibitor (PI3Kdi): *Sell*, *Il7r*, *S1pr1*, *Tcf7*, *Ccr7* and *Klf2*. The relative mRNA abundance is graded from low (blue) to high (red) per row. Input data for the heatmap are listed in **Supplementary Datasheet 3**. **(J, K)** mRNA expression levels (FPKM) of *Ctla4* in **(J)** WT CTL ± PI3K p110δ inhibitor (PI3Kdi) for 24 h and **(K)** in TCR stimulated CTL ± PI3K p110δ inhibitor (PI3Kdi) for 4 h. FPKM shown as the mean of three biological replicates ± standard deviation. **(L)** mRNA expression levels (FPKM) for inhibitory receptors *Pdcd1/*PD-1, *Havcr2*/TIM-3, *Lag3* and *Slamf6* in WT CTL ± PI3K p110δ inhibitor (PI3Kdi). **(D, J, K, L)**: *adj. P <0.05 and fold change >1.5 or <0.67.

In these initial CTL experiments we examined the impact of PI3K p110δ inhibition on an immediate (4 h) response of CTL to TCR engagement. The rationale for this time choice was that CTL activated *via* the TCR undergo activation induced cell death making it difficult to interpret data from longer time points. However, to explore further how PI3K p110δ controls CTL biology, we also examined the impact of sustained inhibition of PI3K p110δ on CTL phenotypes. In this context, CTL maintained in IL-2 have high levels of PtdIns(3,4,5)*P_3_* produced by sustained activity of PI3K p110δ (6, 7). RNA sequencing was thus used to compare the transcriptome of CTL maintained for 24 h in the presence or absence of the PI3K p110δ inhibitor IC87114. In this setting, blocking PI3K p110δ activity did not affect the growth and death of effector CD8^+^ T cells [**Figures 4A, B** and (23)] but did modulate the CTL transcriptome. **Figure 4C** shows increased expression of 254 mRNA and decreased expression of 96 mRNA in CTL lacking PI3K p110δ activity (**Supplementary Table 5** and **Supplementary Datasheet 3**). The mRNA dependent on sustained PI3K p110δ activity in CTL includes chemokines and cytokines (**Figure 4D**). Interestingly, there is no PI3K p110δ regulation of mRNA encoding the costimulatory molecules 4-1BB and ICOS (**Figure 4E**) or mRNA encoding cytolytic effector molecules granzyme and perforin (**Figure 4F**).

The absence of any effect of PI3K p110δ inhibition on expression of cytolytic effector molecules and 4-1BB and ICOS is a fundamental difference between naïve T cells and CTL (**Figures 2H, J** and **Figures 4E, F**). One other difference, in terms of the impact of PI3K p110δ inhibition on TCR responses in naïve T cells versus CTL, is the magnitude of the effects. There are many more TCR regulated transcripts dependent on PI3K p110δ activity in naïve T cells than in CTL (**Figure 4G**).

What are the PI3K p110δ regulated mRNA in CTL? The GO term annotation for mRNA that increased in expression following loss of PI3K p110δ activity in CTL identified molecules linked to the control of cell adhesion and migration, notably CD62L (*Sell*) an adhesion molecule that controls T cell trafficking into secondary lymphoid tissue and *Ccr7* and sphingosine 1-phosphate receptor 1 (*S1pr1*), chemokine receptors that regulates lymphocyte entry and egress from lymphoid tissues (**Figures 4H, I**, and **Supplementary Table 5**). These have previously been described as proteins whose expression is controlled by PI3K p110δ (29, 49). mRNA that increases in expression in CTL treated with the PI3K p110δ inhibitor also include the interleukin 7 receptor (*Il7r*) and the transcription factors *Tcf7 and Klf2* (**Figure 4I** and **Supplementary Table 5**).

Are there any new insights about the role of PI3K p110δ in effector CD8^+^ T cells from these experiments? One fundamental observation is that sustained loss of PI3K p110δ activity in CTL increases mRNA expression of the important inhibitory receptor *Ctla4* (**Figure 4J**). Increased *Ctla4* expression was also seen in TCR activated CTL treated with the PI3K p110δ inhibitor for 4 h (**Figure 4K**). In this respect other critical inhibitory receptors that control CTL function are PD-1, LAG3, TIM3 and SLAMF6: there is no effect of PI3K p110δ inhibition on the mRNA expression of PD-1 or TIM3 but the mRNA expression of LAG3 and SLAMF6 is induced by loss of PI3K p110δ activity (**Figure 4L**). These results inform that the activation of PI3K p110δ represses the expression of three important inhibitory receptors: *Ctla4*, *Slamf6* and *Lag3* in CTL.

### The Contribution of PKB/AKT, FOXO1 and ERK1/2 to the Control of Cytokine and Chemokine Production in CTL

**Figure 5:**
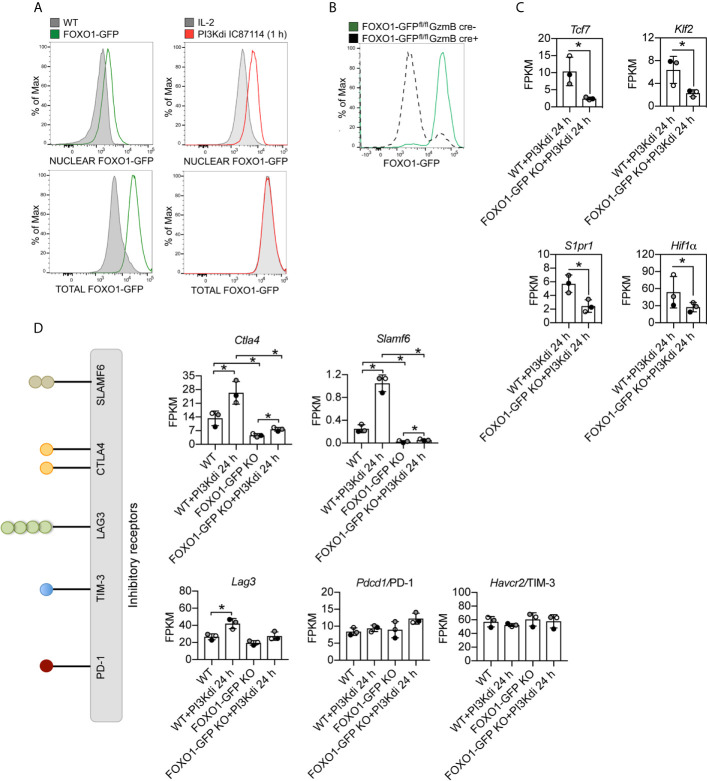
*Ctla4* and *Slamf6* mRNA are modulated by FOXO1 in CTL. FOXO1-GFP null and WT CTL maintained for 24 h in the presence or absence of the PI3K p110δ inhibitor IC87114 (PI3Kdi) were subjected to RNAseq and their transcriptomes compared. **(A)** Nuclear localisation of FOXO1-GFP analysed by flow cytometry in IL-2 maintained CTL and after 1 h treatment with the PI3K p110δ inhibitor. Left panel: purified nuclei from WT CTL (expressing untagged FOXO1) and FOXO1-GFP CTL were compared. The green line indicates detection of FOXO1-GFP expression level in the nucleus (top) or total cell (bottom). Right panel: purified nuclei (top) or total cells (bottom) from FOXO1-GFP CTL either maintained in IL-2 (grey filled line) or treated with PI3K p110δ inhibitor for 1 h (red line) were compared. The right shift of the red line compared to the grey filled line indicates an increased level of FOXO1-GFP in the nuclei of PI3K p110δ inhibitor treated cells (top right), while there is no change in the total FOXO1 protein level (bottom right). Graph of treated cells is representative of three biological replicates. **(B)** Representative histogram showing the deletion of FOXO1-GFP quantified by flow cytometry in CTL generated from FOXO1-GFP^fl/fl^GzmB cre+ (grey dashed line FOXO1-GFP KO) versus FOXO1-GFP^fl/fl^GzmB cre− (green solid line FOXO1-GFP) mice. **(C)** mRNA expression levels (FPKM) of *Tcf7*, *Klf7*, *S1pr1* and *Hif1α* in WT and FOXO1-GFP KO CTL lacking PI3K p110δ activity. **(D)** Histograms showing mRNA expression levels (FPKM) for inhibitory receptors *Ctla4*, *Slamf6*, *Lag3*, *Pdcd4/*PD-1 and *Havcr2/*TIM3 in WT and FOXO1-GFP KO CTL ± PI3K p110δ inhibitor (PI3Kdi). **(C, D)** FPKM shown as the mean of three biological replicates ± standard deviation. *adj. P <0.05 and fold change >1.5 or <0.67.

**Figure 6:**
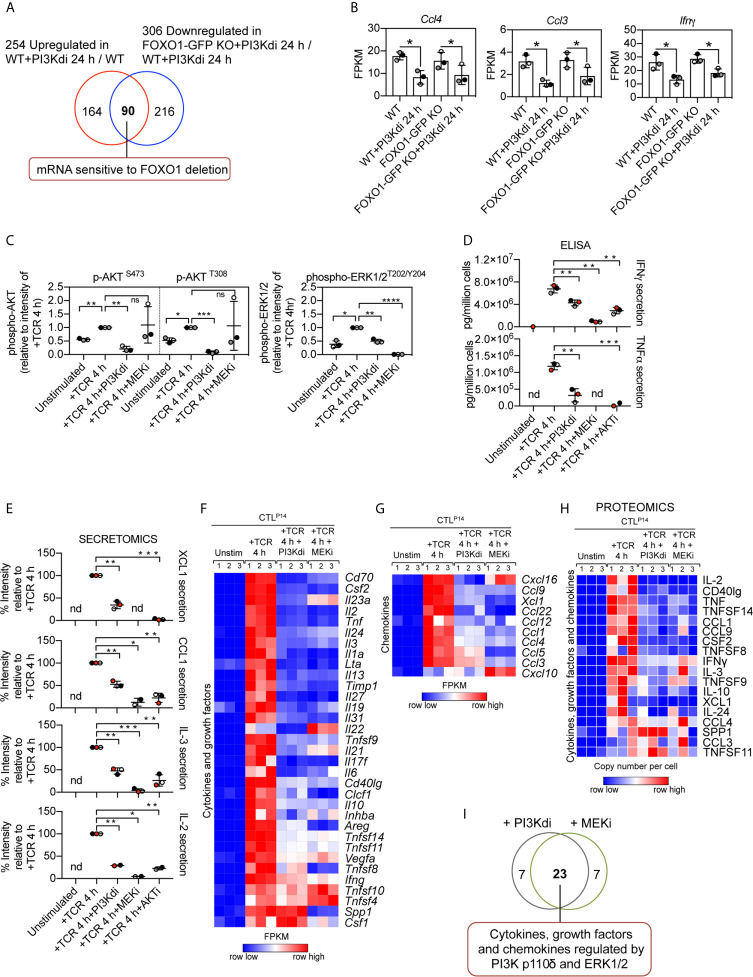
Contribution of PKB/AKT, FOXO1 and ERK1/2 to the control of cytokine and chemokine production in CTL. **(A)** Venn diagram showing the overlap in the number of mRNA whose expression was induced by sustained PI3K p110δ inhibition in WT CTL and reduced in treated FOXO1-GFP KO CTL versus WT treated CTL. **(B)** mRNA expression levels (FPKM) of *Ccl4*, *Ccl3* and *Ifnγ* in WT and FOXO1-GFP KO CTL ± PI3K p110δ inhibitor. *adj. P <0.05 and fold change >1.5 or <0.67. **(C)** Quantification of phosphorylated AKT (S473 and T308) and ERK1/2 (T202/Y204) in CTL in response to TCR stimulation in the presence or absence of PI3K p110δ (PI3Kdi) or MEK (MEKi) inhibitor assayed by western blot (**Supplementary Figure 7A**). **(D)** Levels of Interferon gamma (IFNγ) and Tumour necrosis factor alpha (TNFα) present in the supernatant of P14 CTL unstimulated and TCR retriggered in the presence or absence of either PI3K p110δ (PI3Kdi), MEK (MEKi) or AKT (AKTi) inhibitor determined by ELISA. **(E)** Levels of XCL1, CCL1, IL-3 and IL-2 protein secreted in the supernatant of unstimulated, TCR stimulated and TCR stimulated CTL in the presence of either PI3K p110δ (PI3Kdi), MEK (MEKi) or AKT (AKTi) inhibitor assayed by mass spectrometry. Transcriptome and proteome of P14 CTL unstimulated and TCR retriggered in the presence or absence of either PI3K p110δ (PI3Kdi) or MEK (MEKi) inhibitor were characterised by RNAseq and mass spectrometry respectively. Impact of PI3K p110δ and ERK1/2 inhibition on TCR induced cytokines and chemokines mRNA and protein expression was assessed. **(F, G)** Heatmaps showing mRNA encoding for annotated **(F)** cytokines, growth factors and **(G)** chemokines (Cytokine activity GO:0005125 and Chemokine activity GO:0008009). **(H)** Heatmap of proteins annotated as cytokines, growth factors and chemokines. Heatmaps show the relative mRNA abundance or the relative protein abundance graded from low (blue) to high (red) per row. Input data for heatmaps are listed in **Supplementary Datasheets 2** **(F, G)** and **4** **(H)**. **(I)** Overlap in the number of cytokine, growth factor and chemokine mRNA regulated by both PI3K p110δ and ERK1/2. Data in **(C–E)** shown as the mean of three biological replicates ± standard deviation and statistical analysis is calculated by unpaired, unequal variance t-test with Welch’s correction. The P values are considered as follow: *P <0.05, **P <0.01, ***P <0.001, ****P <0.0001. 'ns' not statistically significant.

One major role for PI3K p110δ in CTL is to control the nuclear localisation of the transcription factor FOXO1. CTL have high levels of PtdIns(3,4,5)*P_3_* which drives phosphorylation and activation of the serine/threonine kinase AKT which phosphorylates FOXO1 causing its nuclear exclusion. Inhibition of PI3K p110δ, which prevents PtdIns(3,4,5)*P_3_* production and AKT phosphorylation, causes a rapid translocation of FOXO1 to the nucleus [**Figure 5A** and (6)]. Here, a pertinent point is that the PI3K p110δ repression of CD62L, CCR7, S1P1R, IL7R, TCF7 and KLF2 is mediated by FOXO1 (29, 49). A key question then is whether the repressive effect that PI3K p110δ activity has on the expression of *Ctla4*, *Slamf6* and *Lag3* mRNA in CTL is mediated by FOXO1. In this context, it has been described that the expression of PD-1 in CD8^+^ T cells is FOXO1 mediated (28) but the role of FOXO1 in controlling expression of other inhibitory receptors in CTL has not been examined.

To explore the mechanisms used by PI3K p110δ to control T cells we analysed the impact of PI3K p110δ inhibition on the expression of mRNA encoding CTLA4, SLAMF6, PD-1, TIM-3 and LAG3 in FOXO1 null CTL (FOXO1-GFP KO). To delete *Foxo1* in CTL we backcrossed mice with floxed *Foxo1*-*EGFP* alleles (32) to transgenic mice expressing Cre recombinase under the control of a granzyme B promoter that allows deletion of FOXO1-GFP in CTL (**Figure 5B**, **Supplementary Figures 5A, B**). In normal CTL >90% of FOXO1 is excluded from the nucleus and hence is transcriptionally inactive, whereas in CTL treated with the PI3K p110δ inhibitor FOXO1 relocates to the nucleus. We therefore performed RNA sequencing of FOXO1-GFP null CTL before and after 24 h treatment with the PI3K p110δ inhibitor and compared to WT treated CTL (**Supplementary Datasheet 3**). The full list of FOXO1 dependent mRNA identified in these experiments is shown in supplemental data (**Supplementary Table 6**) and includes previously identified FOXO1 targets including *Tcf7*, *Klf2* and *S1pr1* and as well as the transcription factor Hif1a (**Figure 5C**). There were also mRNAs whose expression was enhanced in FOXO1 null CTL (**Figure 5C**). These data also show that the expression of *Ctla4* and *Slamf6* mRNA is dependent on FOXO1 (**Figure 5D**). In this respect, recent chromatin analysis (ChIP-seq) has identified FOXO1 binding to *Ctla4* and *Slamf6* gene loci and ATAC-seq analytic comparisons of wild type and FOXO1 null CD8^+^ T cells have indicated reduced chromatin accessibility in the *Ctla4* and *Slamf6* gene loci in FOXO1 null CD8^+^ T cells [**Supplementary Figure 6A** and (50)].

Interestingly, not all inhibitory receptor mRNA expression was changed in FOXO1 null CTL. **Figure 5D** shows *Lag3* mRNA is still expressed in FOXO1 null CTL, though it is marginally lower, and PD-1 and TIM-3 are not regulated by PI3K p110δ or FOXO1 (**Figure 5D**). It is also pertinent that not all the PI3K p110δ regulated mRNA in CTL are sensitive to FOXO1 deletion (**Figure 6A**; **Supplementary Table 7** and **Supplementary Datasheet 3**). For example, inhibition of PI3K p110δ in CTL causes decreased expression of chemokine (*Ccl3, Ccl4*) and cytokine (*Ifnγ*) mRNA in both WT and FOXO1 null T cells (**Figure 6B**). In this respect, previous studies have shown that the nuclear exclusion of FOXOs is necessary for the production of Interferon gamma (23), which would be consistent with a repressive role for FOXO1 in the control of Interferon gamma production. The current data found no evidence for enhanced expression of Interferon gamma mRNA in FOXO1 null CTL (**Figure 6B**), which highlights that the regulation of this key cytokine is not controlled by a simple FOXO1 dependent switch but requires co-ordination with other signalling pathways. It also argues that the profound effect of PI3K p110δ on Interferon gamma production is not mediated solely by PI3K p110δ control of FOXOs. In this respect, CTL lacking PI3K p110δ activity show reduced activation of the MAP kinases ERK1 and ERK2 in response to antigen receptor engagement [**Figure 6C**, **Supplementary Figure 7A** and (29, 30)]. This is germane because the activity of ERK1/2 is necessary for cytokine and chemokine production by antigen activated naïve CD8^+^ T cells (51). We therefore evaluated the PKB/AKT and ERK1/2 signalling requirements of antigen receptor induced cytokine and chemokine production in CTL. The data show that TCR induced production of multiple chemokines and cytokines is dependent on both the activation of the PKB/AKT serine threonine kinases and ERK1/2 activity (**Figures 6D, E**). In particular the PI3K p110δ controlled cytokines and chemokines mRNA, shown in **Figures 3G, H** and protein in **Figure 3I**, are also ERK1/2 controlled (**Figures 6F–I**, **Supplementary Figure 8A**, **Supplementary Tables 8**, **9** and **Supplementary Datasheets 2**, **4**). These data collectively highlight that PI3K p110δ control of the CD8^+^ T cell transcriptional programs reflects the role of this lipid kinase in co-ordinating the activity of multiple signalling pathways.

## Discussion

The aim of the present study was to gain an in-depth knowledge of the contribution of PI3K p110δ to antigen receptor controlled programs in naïve and effector CD8^+^ T cells to understand how this lipid kinase controls CD8^+^ T cell differentiation. One important conclusion is that the dominant targets for PI3K p110δ regulation are the secreted molecules and cell surface receptors that mediate CD8^+^ T cells communication with other immune cells. The production of multiple cytokines by TCR activated CD8^+^ T cells thus requires PI3K p110δ activity. Notable amongst these are cytokines that control the activation of monocytes and macrophages such as GM-CSF/CSF2, TNF and IFNγ. One other important insight is that PI3K p110δ controls the production of chemokines mRNA which orchestrate the ability of CD8^+^ T cells to recruit innate immune cells to sites of infection (45, 52).

PI3K p110δ activity also selectively shapes the repertoire of costimulatory receptor that control CD8^+^ T cell differentiation. For example, in naïve CD8^+^ T cells, TCR induced upregulation of the mRNA encoding the costimulatory receptor 4-1BB, which is essential for the persistence of tissue resident CD8^+^ effector/memory T cells (53–55), is dependent on PI3K p110δ activity. Further support for the concept that PI3K p110δ controls the ability of CD8^+^ T cells to communicate with other immune cells includes the role of PI3K p110δ in controlling expression of the inhibitory receptors *Ctla4* and *Slamf6* mRNA in effector CTL. Hence the loss of PI3K p110δ activity and the relocalisation of the transcription factor FOXO1 to the nucleus upregulates *Ctla4* and *Slamf6* mRNA expression. The significance of this result stems from the essential role that CTLA4 and SLAMF6 have as immunomodulatory/inhibitory receptors that restrain peripheral T cell responses to maintain peripheral tolerance (56–58). In this context, an elegant study by Kaech and colleagues has shown that FOXO1 is necessary for the differentiation of PD-1^hi^ Eomes^hi^ terminally exhausted CTL. Moreover, FOXO1 null cytotoxic T cells fail to persist and control chronic viral infection (28). The authors hypothesised that the importance of FOXO1 in controlling T cell exhaustion reflected that FOXO1 controlled expression of the inhibitory receptor PD-1 (28). The current study forces a revision of this conclusion by demonstrating that FOXO1 does not directly control PD-1 expression in CD8^+^ T cells. Hence inhibition of PI3K p110δ, which immediately causes FOXO1 to relocalise to the nucleus to transcriptionally reprogram CTL, does not impact PD-1 expression. Moreover, there is no loss of PD-1 expression in FOXO1 null CD8^+^ T cells. The expression of inhibitory receptors is thus not a simple on/off FOXO1 switch but requires integration of multiple signals with evidence that the oxygen environment is particularly important (59). In terms of T cell exhaustion and the ability of PI3K p110δ to control CD8^+^ effector/memory T cells transition then FOXO1 control of *Ctla4* and *Slamf6* mRNA could be relevant as these two molecules control T cell exhaustion (26, 60, 61). However, the role of FOXO1 in enforcing T cell quiescence by controlling expression of *Tcf7*, *Klf2* and AP-1 family transcription factors will also be important in regulating the effector/memory versus senescent fate of CD8^+^ T cells (50). Moreover, PI3K p110δ and FOXO1 control of T cell trafficking, by regulating the expression of key adhesion receptors and chemokine receptors, will also influence CD8^+^ T cell immune responses *in vivo* as will PI3K p110δ control of the production of inflammatory cytokines and chemokines that recruit and/or regulate innate immune cells.

One salient insight from the current work is the magnitude and selectivity of the effects of PI3K p110δ on the transcriptional restructuring that accompanies T cell differentiation. Hence only 6% of the TCR regulated transcripts in naïve T cells were sensitive to loss of PI3K p110δ activity and in CTL this dropped to 3%.

One notable result was that expression of the major regulatory components of the metabolic programs that control T cell growth and differentiation are not dependent on PI3K p110δ activation. In this respect, T cell metabolic programs are controlled by the transcription factor Myc and serine/threonine kinase mTORC1 (mammalian target of rapamycin complex 1) (41, 48). We have shown previously that PI3K p110δ activation is not required for mTORC1 activity in CD8^+^ T cells (29, 43) and the present data found no role for PI3K p110δ in Myc mRNA expression or in controlling expression of Myc targets. Nor did PI3K p110δ prevent expression of *Hif1α* mRNA in CD8^+^ T cells. In this respect, analysis of the impact of mTORC1 inhibition of CTL transcriptome identified mRNA encoding glycolytic regulators and molecules that control lipid metabolism and ribosome biogenesis as major targets for mTORC1 signalling pathways (7) whereas the data herein show these were not regulated directly by PI3K p110δ. It should be emphasised there are some common targets for mTORC1 and PI3K p110δ notably KLF2 and receptors such as CD62L and S1PR1 that control T cell trafficking (7, 29, 43). The existence of these shared targets has historically been explained using a linear model whereby PI3K p110δ controls T cell responses *via* mTORC1 (29). However, it is now recognised that a shared requirement for PI3K p110δ and mTORC1 activity rather reflects that these signalling molecules control pathways that can converge on a single target (43). Indeed, one key observation herein is that even the role of PI3K p110δ in controlling T cell transcriptomes cannot be ascribed to a simple linear signalling pathway. For example, the ability of PI3K p110δ to control the nuclear exclusion of FOXO1 is critical for T cell differentiation yet PI3K p110δ and PKB/AKT dependent but FOXO1 independent mechanisms are also relevant. As well the ability of PI3K p110δ to repress the activity of ERKs can also contribute to its ability to modulate T cell differentiation.

Finally, a fundamental finding from the present study was that there are differences in how PI3K p110δ inactivation impacts T cell antigen receptor activated lymphocytes as they exit quiescence versus effects in effector T cells. Effector CTL are thus far less sensitive to loss of PI3K p110δ activity than naïve T cells. Moreover, inhibition of PI3K p110δ could affect the expression of cytolytic effector molecules such as the granzymes and perforin in TCR activated naïve CD8^+^ T cells but not in CTL. PI3K p110δ inhibition could also selectively suppress the production of cytokines and chemokines in both naïve T cells and CTL but did not prevent degranulation and release of cytolytic molecules by CTL. These results are pertinent because when thinking of how to use PI3K p110δ inhibitors therapeutically it is important to consider whether the objective is to supress the effector immune response or to prevent *de novo* immune activation. Many screens focus on how inhibitors prevent primary activation of quiescent peripheral blood derived T cells. The current study highlights how unbiased screening of both naïve and effector populations would give a better perspective on the potential therapeutic benefit of signalling inhibitors.

## Data Availability Statement

The datasets presented in this study can be found in online repositories. The names of the repository/repositories and accession number(s) can be found below: https://www.ncbi.nlm.nih.gov/geo/, GSE169436; https://www.ncbi.nlm.nih.gov/geo/, GSE169449; https://www.ncbi.nlm.nih.gov/geo/, GSE169572; http://www.proteomexchange.org/, PXD025046; http://www.proteomexchange.org/, PXD025061. ChIP-seq, and ATAC-seq datasets were previously published (50) and deposited to the NCBI GEO/SRA and can be accessed with the identifier GSE163723.

## Ethics Statement

The animal study was reviewed and approved by the University Ethical Review Committee under the authorisation of the UK Home Office Animals (Scientific Procedures) Act 1986.

## Author Contributions

LS and DC designed the experiments. LS, AN, and MD performed the experiments. JM did the bioinformatics for the RNAseq data. LS analysed the data and produced the figures. LS and DC interpreted the results and wrote the manuscript. DC conceived the study, supervised the experiments and provided financial support. All authors contributed to the article and approved the submitted version.

## Funding

This research was supported by a Wellcome Trust Principal Research Fellowship to DC (205023/Z/16/Z). JM was supported by an Australian NHMRC CJ Martin Early Career fellowship and JM and DC have received funding from the European Union’s Horizon 2020 research and innovation programme under the Marie Sklodowska Curie grant agreement No. 705984. AN was supported by the MRC doctoral training program (MR/K501384/1).

## Conflict of Interest

The authors declare that the research was conducted in the absence of any commercial or financial relationships that could be construed as a potential conflict of interest.

## Supplementary Material

Supplementary Data Sheet 1 CD8 naive and TCR activated 24 h ± inhibitor RNAseq data.

Supplementary Data Sheet 2 Unstim and TCR stimulated 4 h CTL ± inhibitors RNAseq data.

Supplementary Data Sheet 3 WT and FOXO1-GFP KO CTL ± inhibitor 24 h RNAseq data.

Supplementary Data Sheet 4 Unstim and TCR stimulated 4 h CTL ± inhibitors proteomic data.

Supplementary Table 1 mRNA changing significantly in naïve CD8+ T cells after 24 h TCR activation.

Supplementary Table 2 mRNA changing significantly in 24 h TCR activated cells after PI3K p110δ inhibition.

Supplementary Table 3 mRNA changing significantly in CTL after 4 h TCR retriggering.

Supplementary Table 4 mRNA changing significantly in 4 h TCR retriggered CTL after PI3K p110δ inhibition.

Supplementary Table 5 mRNA changing significantly in WT CTL after 24 h PI3K p110δ inhibition.

Supplementary Table 6 mRNA changing significantly in PI3K p110δ treated FOXO1-GFP KO versus WT treated CTL.

Supplementary Table 9 mRNA changing significantly in 4 h TCR retriggered CTL after ERK1-2 inhibition.
