# Supplementary material for: Phosphoinositide 3-Kinase p110 Delta Differentially Restrains and Directs Naïve Versus Effector CD8+ T Cell Transcriptional Programs
Source: Front Immunol. 2021 Jun 18;12:691997. doi: 10.3389/fimmu.2021.691997 (PMC8250422; doi:10.3389/fimmu.2021.691997)
Supplement: Supplementary Data Sheet 1 — CD8 naive and TCR activated 24 h ± inhibitor RNAseq data. [file DataSheet_1.zip › Data sheet1/Supplementary Figures and Table 7 and 8.docx]

**Supplementary Figure 1**


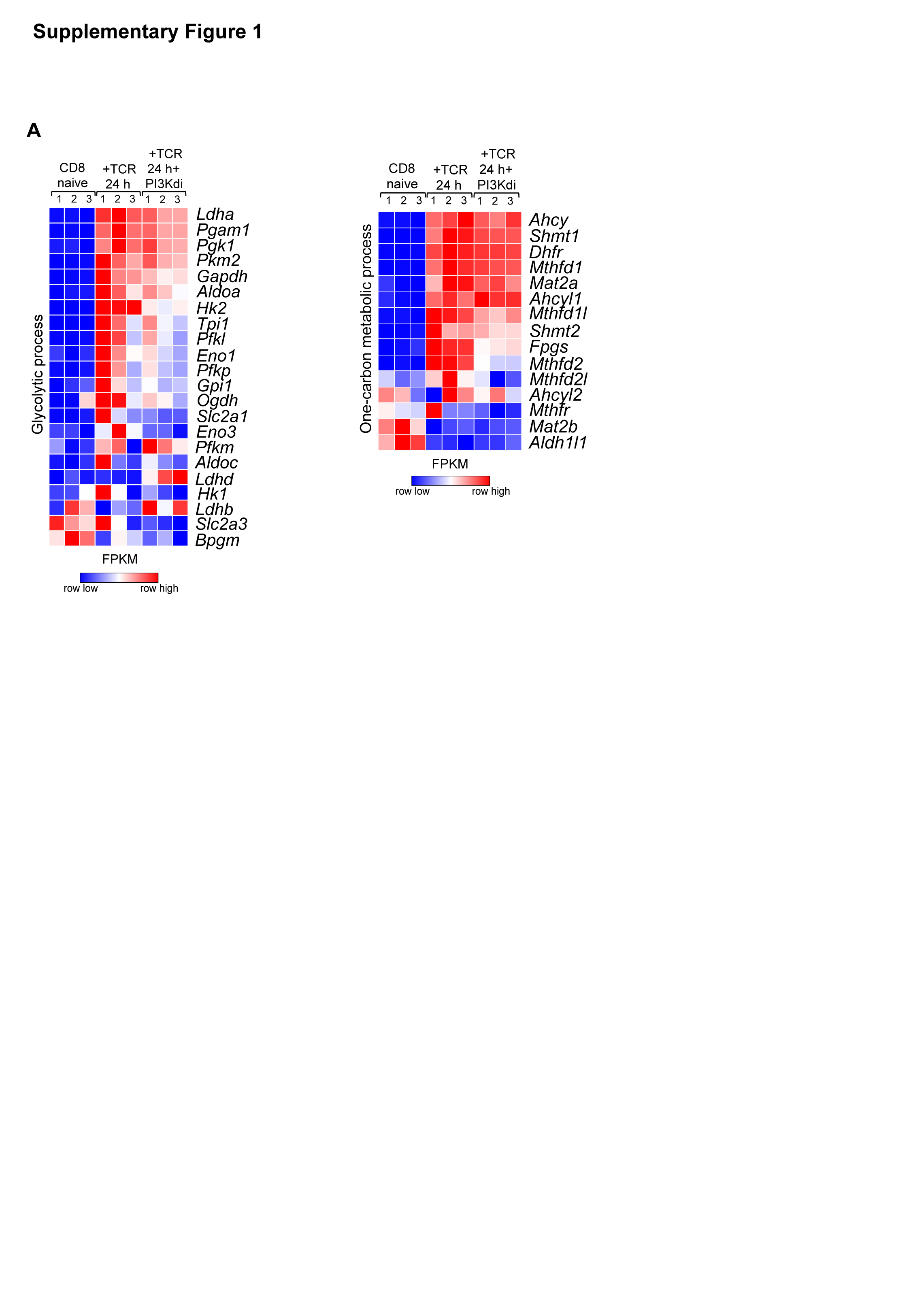


**Supplementary Figure 1 TCR induced changes in expression of mRNA encoding metabolic machinery enzymes are not PI3K p110δ regulated in activated CD8^+^ T cells.** Heatmaps of mRNA encoding enzymes that mediate glycolysis (Glycolytic process GO:0006096) and One-carbon metabolism (One-carbon metabolic process GO:0006730). Heatmaps show the relative mRNA abundance graded from low (blue) to high (red) per row. Input data for heatmaps are listed in Supplementary Datasheet 1**.**

**Supplementary Figure 2**


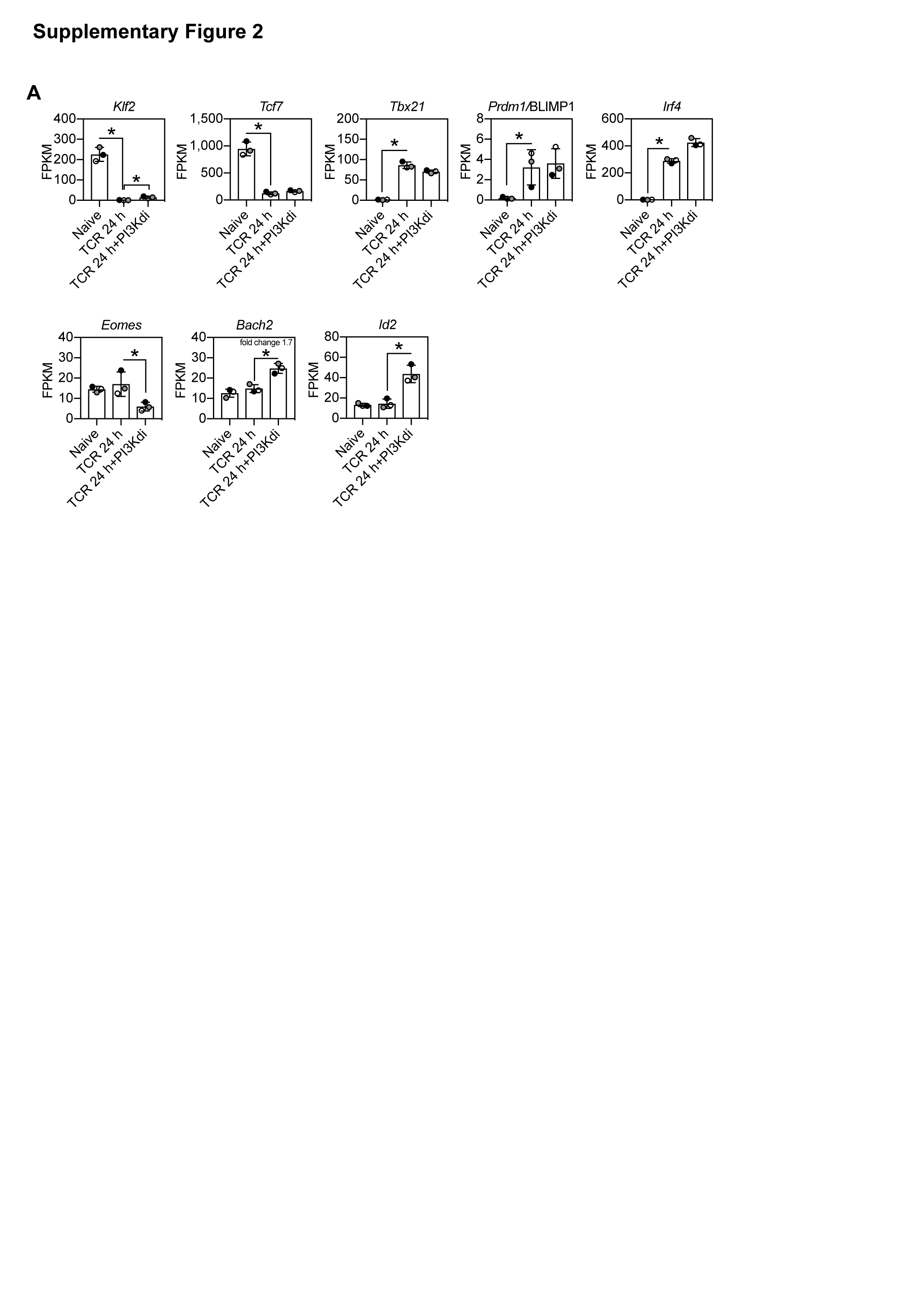


**Supplementary Figure 2 Impact of PI3K p110δ inhibition on the expression of mRNA encoding transcription factors key for the regulation of CD8^+^ T cell differentiation.** mRNA expression levels (FPKM) of key transcription factors shown as the mean of 3 biological replicates ± standard deviation.

**Supplementary Figure 3**


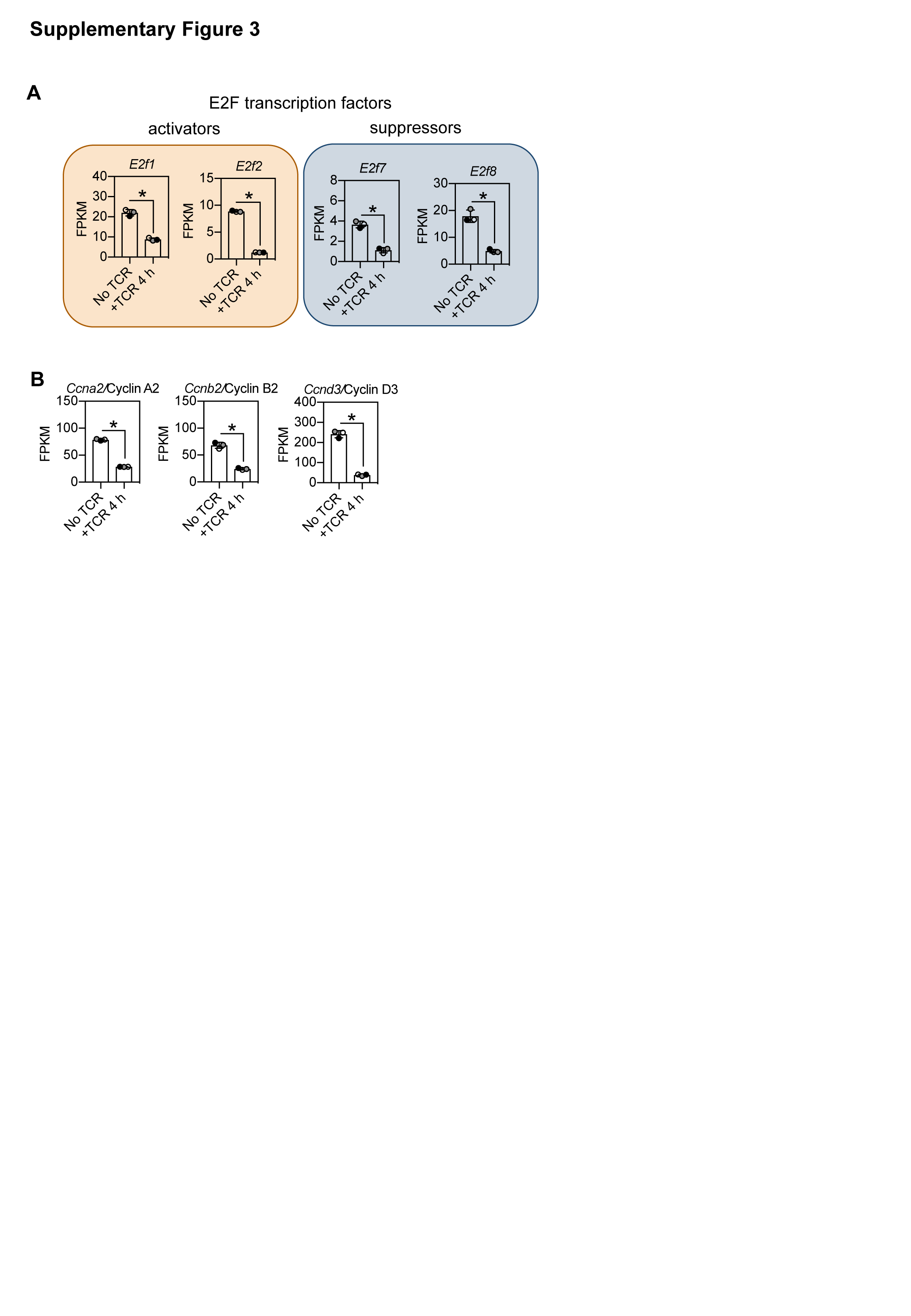


**Supplementary Figure 3 Antigen receptor regulation of cell cycle mRNA expression in effector CD8^+^ T cells.** mRNA expression levels (FPKM) of **(A)** E2F transcription factors: *E2f1*, *E2f2*, *E2f7* and *E2f8*, and **(B)** Cyclins: *Ccna2*/Cyclin A2, *Ccnb2*/Cyclin B2 and *Ccnd3*/Cyclin D3. FPKM shown as the mean of 3 biological replicates ± standard deviation.

**Supplementary Figure 4**


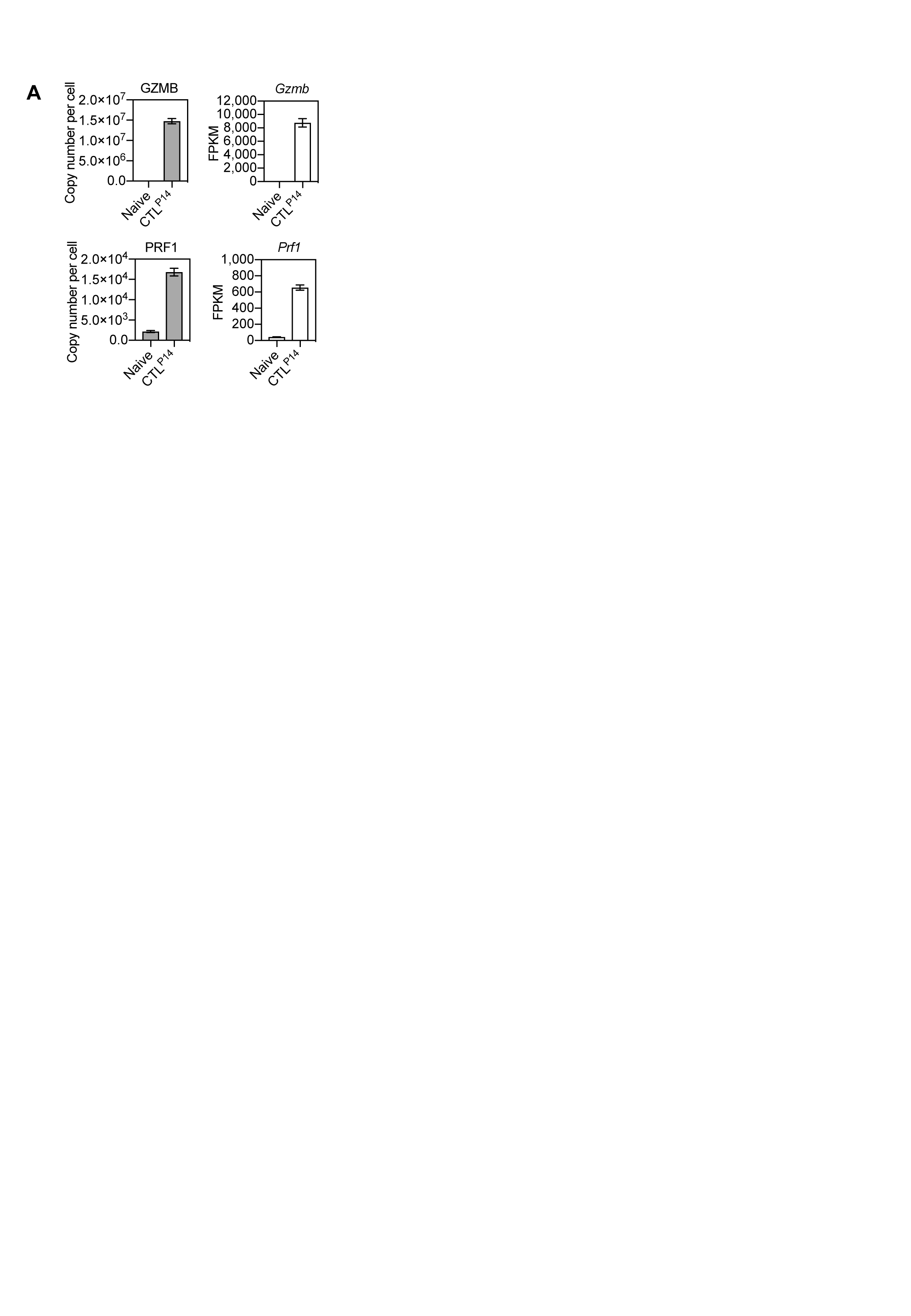


**Supplementary Figure 4 mRNA and protein expression profile of effector molecules in naïve and effector CD8^+^ T cells. (A)** Expression levels of granzyme B protein (copy number per cell) (Howden et al., 2019 and ImmPRes http://immpres.co.uk) or mRNA (FPKM) in naïve CD8^+^ T cells and CTL. Copy numbers and FPKM shown as the mean of 3 biological replicates ± standard deviation.

**Supplementary Figure 5**

**
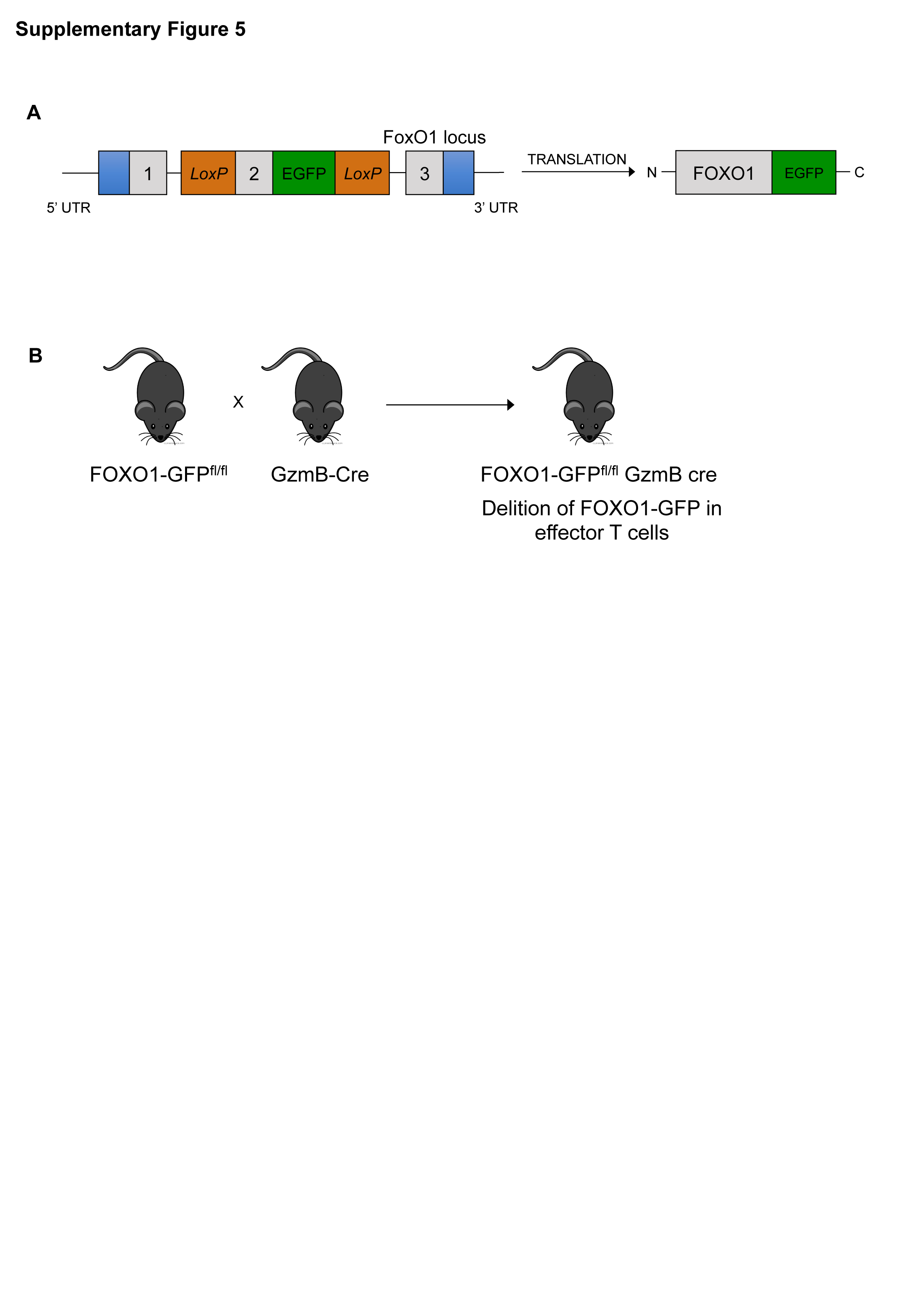
**

**Supplementary Figure 5 Generation of a mouse with a floxed *Foxo1-EGFP* gene and conditional deletion in activated T cells with Granzyme B cre. (A)** Schematic of floxed *Foxo1-EGFP*. EGFP sequence is inserted between the codon coding for the last amino acid of FOXO1 and the stop codon in exon 2 of the *Foxo1* gene. This enables the translation of FOXO1-GFP fusion protein. Exon 2, which includes the EGFP sequence, is flanked by LoxP sites allowing conditional deletion. **(B)** Schematic diagram of generating a conditional knockout mouse for the deletion of *Foxo1-EGFP* gene in activated T cells. FOXO1-GFP^fl/fl^ GzmB cre mice were generated by crossing floxed *Foxo1-EGFP* mice to mice expressing the Cre recombinase under the control of the granzyme B promoter sequence.

**Supplementary Figure 6**

**
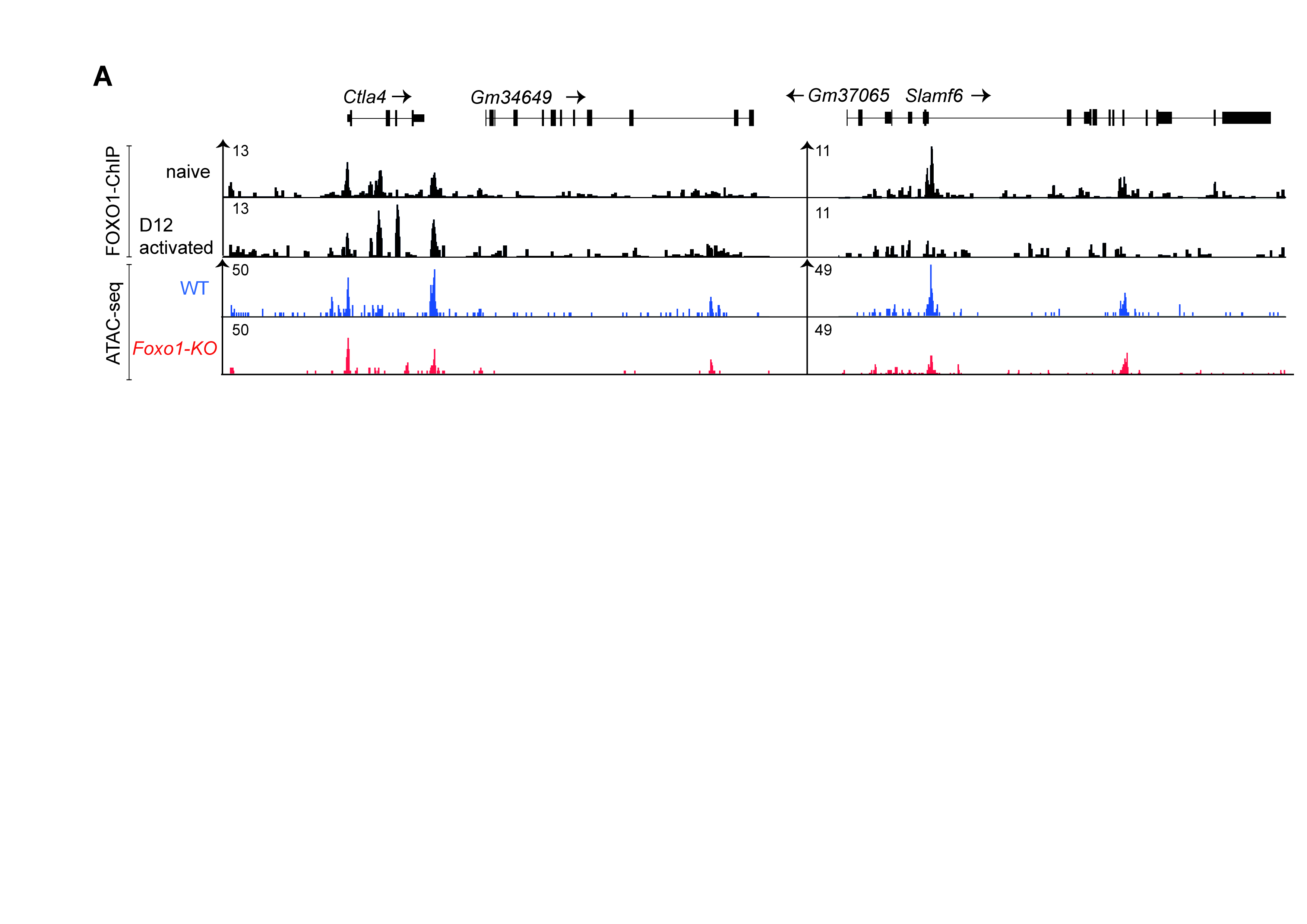
**

**Supplementary Figure 6 Direct FOXO1 regulation of *Ctla4* and *Slamf6*. (A)** FOXO1 genomic binding (ChIP-seq) in naive and D12 post-infection (LCMV-ARM) P14 CD8^+^ T cells, and chromatin accessibility (ATAC-seq) in naïve WT and FOXO1 KO CD8^+^T cells for *Ctla4* and *Slamf6* from published datasets (1) deposited to the NCBI GEO/SRA and accessible with the identifier GSE163723. IGV genome browser ((2) version 2.8.10) was used to visualise ChIP and ATAC peaks on the mouse genome (mm10).

**Supplementary Figure 7**

**
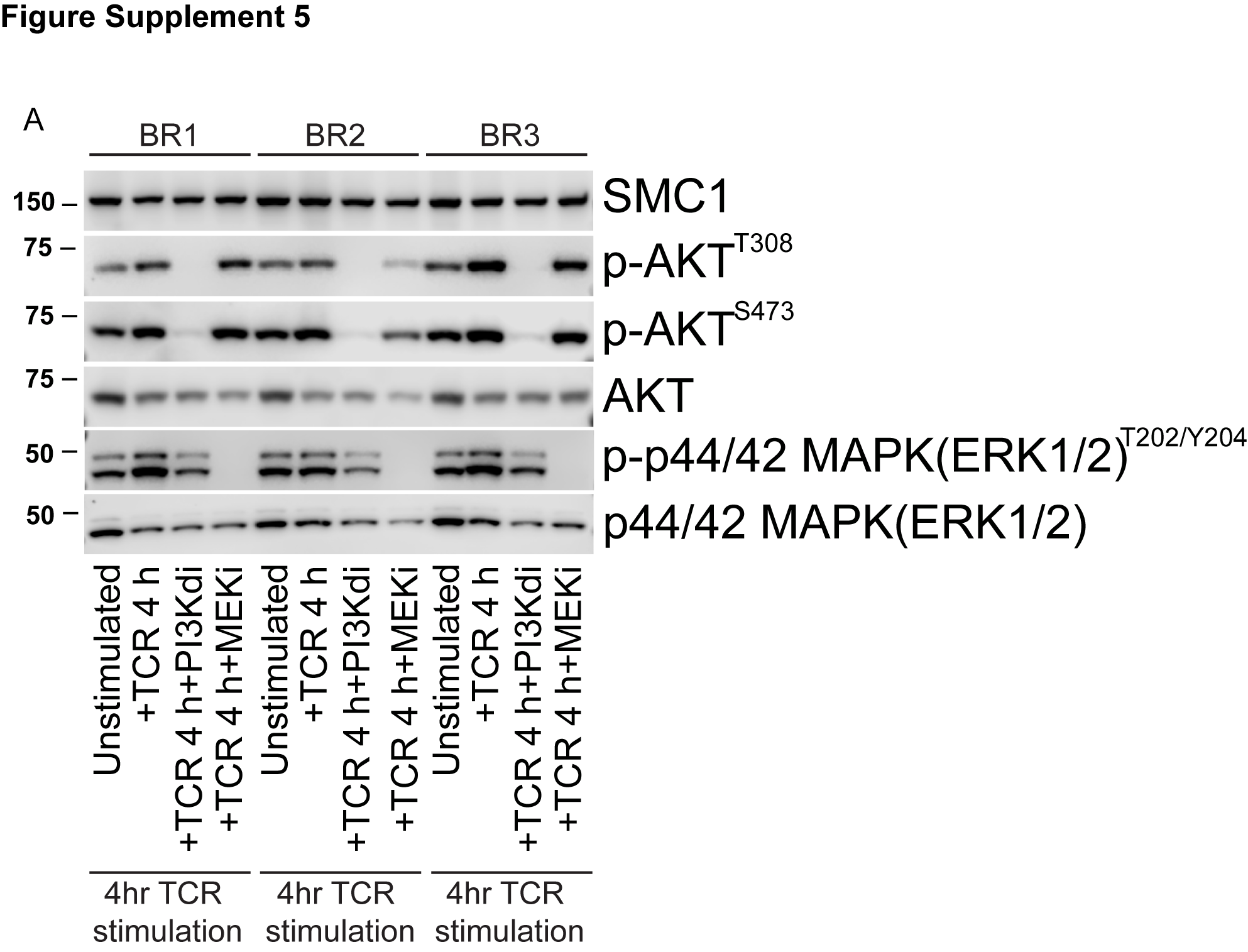
**

**Supplementary Figure 7 The selective PI3K p110δ inhibitor IC87114 and MEK inhibitor PD184252 allow effective inhibition of AKT and ERK1/2 phosphorylation respectively in effector CD8^+^ T cells. (A)** Effector CD8^+^ T cells were left unstimulated or TCR retriggered with gp33-41 peptide ± PI3K p110δ inhibitor IC87114 (PI3Kdi) or MEK inhibitor PD184252 (MEKi) for 4 h. Cell lysates were analysed by western blot using phospho-AKT antibodies (p-AKT S473 and T308), phospho-ERK1/2 (p-p44/42 MAPK T202/Y204), AKT, ERK1/2 (p44/42 MAPK) and SMC1 as loading control.

**Supplementary Figure 8**

**
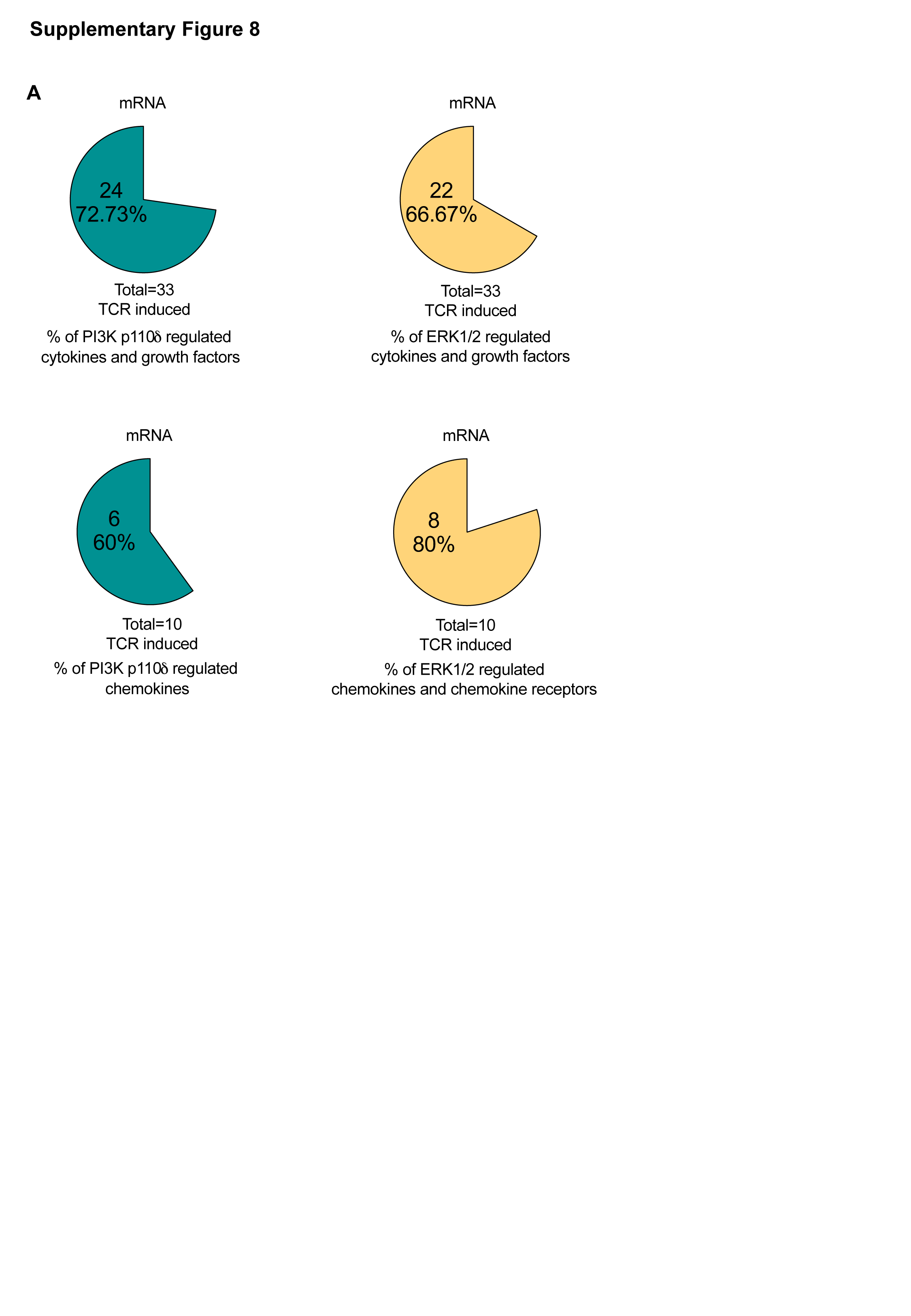
**

**Supplementary Figure 8 Cytokine, growth factor and chemokine mRNA regulated by PI3K p110δ and/or ERK1/2. (A)** The number of cytokine, growth factor and chemokine mRNA PI3K p110δ and/or ERK1/2 regulated is presented as proportion of the total number of cytokines, growth factors and chemokines annotated and TCR induced.

**Supplementary Table 7:** **mRNA sensitive to FOXO1 deletion.** Overlap of mRNA whose expression was statistically significant induced by sustained PI3K p110δ inhibition in WT CTL and reduced in treated FOXO1-GFP KO CTL versus WT treated CTL (fold change > 1.5 or < 0.67; adj. *P* values < 0.05). The shared mRNA listed in the table are therefore regulated by PI3K p110δ through FOXO1.

| **FOXO1 dependent mRNA** | | | |
| --- | --- | --- | --- |
| **Gene symbol** | **Fold change**  **FOXO1-GFP KO +PI3Kdi 24 h / WT +PI3Kdi 24 h** | **Gene symbol** | **Fold change**  **FOXO1-GFP KO +PI3Kdi 24 h / WT +PI3Kdi 24 h** |
| \| *Slamf6* \| \| --- \| \| *Tbc1d4* \| \| *Aff3* \| \| *Aqp3* \| \| *Tnfrsf23* \| \| *Serpinb1a* \| \| *A630023P12Rik* \| \| *Mmp9* \| \| *Tnfrsf22* \| \| *Arap3* \| \| *Gm12349* \| \| *Jchain* \| \| *Ust* \| \| *Prss12* \| \| *Tcf7* \| \| *Dnah12* \| \| *Trat1* \| \| *Matn2* \| \| *Gucy2e* \| \| *Slc28a2b* \| \| *Ripor2* \| \| *Gm11346* \| \| *Ctla4* \| \| *Lyst* \| \| *Lypd6b* \| \| *Igf1r* \| \| *Fbln1* \| \| *Pim2* \| \| *Gpld1* \| \| *Itga4* \| \| *Abcc3* \| \| *Tmem71* \| \| *Ly6g* \| \| *Il1rl2* \| \| *Dgka* \| \| *Ttyh3* \| \| *Klf2* \| \| *Serpina3g* \| \| *Ifi209* \| \| *Gch1* \| \| *S1pr1* \| \| *Pdzd2* \| \| *Xdh* \| \| *Lmtk3* \| \| *Actr3b* \| | \| 0.05 \| \| --- \| \| 0.11 \| \| 0.12 \| \| 0.14 \| \| 0.15 \| \| 0.16 \| \| 0.18 \| \| 0.19 \| \| 0.19 \| \| 0.20 \| \| 0.20 \| \| 0.20 \| \| 0.21 \| \| 0.22 \| \| 0.22 \| \| 0.23 \| \| 0.25 \| \| 0.25 \| \| 0.25 \| \| 0.27 \| \| 0.28 \| \| 0.28 \| \| 0.29 \| \| 0.31 \| \| 0.31 \| \| 0.32 \| \| 0.32 \| \| 0.33 \| \| 0.33 \| \| 0.33 \| \| 0.33 \| \| 0.34 \| \| 0.34 \| \| 0.35 \| \| 0.38 \| \| 0.38 \| \| 0.39 \| \| 0.39 \| \| 0.39 \| \| 0.40 \| \| 0.40 \| \| 0.41 \| \| 0.41 \| \| 0.42 \| \| 0.42 \| | \| *Sh3bp5* \| \| --- \| \| *Tnfrsf26* \| \| *Csgalnact1* \| \| *Plekhg2* \| \| *Selenop* \| \| *Gm13546* \| \| *Pik3r5* \| \| *Ssh2* \| \| *Myof* \| \| *Abhd15* \| \| *Dst* \| \| *Fam49a* \| \| *Tbxa2r* \| \| *Ssbp2* \| \| *Gab3* \| \| *C030034L19Rik* \| \| *Pde4b* \| \| *Palm* \| \| *Ifi214* \| \| *Slc26a11* \| \| *Card6* \| \| *Cxcr4* \| \| *Bach2* \| \| *Lrrc32* \| \| *Kcnj8* \| \| *Phlpp1* \| \| *Ltb* \| \| *Fkbp1a* \| \| *Ifi206* \| \| *Calhm6* \| \| *Gbp2* \| \| *Ifit1bl1* \| \| *Acvr2a* \| \| *Bcl2l11* \| \| *Plxdc2* \| \| *Cmah* \| \| *Slfn1* \| \| *Prnp* \| \| *Dgat1* \| \| *Pdcd4* \| \| *Tmem35b* \| \| *Gm8369* \| \| *Rasa3* \| \| *Tiparp* \| \| *Neurl3* \| | \| 0.42 \| \| --- \| \| 0.43 \| \| 0.43 \| \| 0.44 \| \| 0.44 \| \| 0.44 \| \| 0.45 \| \| 0.45 \| \| 0.46 \| \| 0.47 \| \| 0.49 \| \| 0.49 \| \| 0.49 \| \| 0.49 \| \| 0.50 \| \| 0.50 \| \| 0.51 \| \| 0.52 \| \| 0.53 \| \| 0.53 \| \| 0.53 \| \| 0.54 \| \| 0.55 \| \| 0.56 \| \| 0.56 \| \| 0.56 \| \| 0.56 \| \| 0.57 \| \| 0.57 \| \| 0.58 \| \| 0.59 \| \| 0.60 \| \| 0.61 \| \| 0.61 \| \| 0.61 \| \| 0.61 \| \| 0.63 \| \| 0.63 \| \| 0.64 \| \| 0.64 \| \| 0.64 \| \| 0.65 \| \| 0.65 \| \| 0.66 \| \| 0.67 \| |

**Supplementary Table 8:** **Cytokine, growth factor and chemokine mRNA regulated by both PI3K p110δ and ERK1/2.** List of annotated and TCR induced mRNA encoding for cytokines, growth factors and chemokines whose expression was reduced by blocking either PI3K p110δ or ERK1/2.

| **Cytokines, growth factors and chemokines regulated by PI3K p110δ and ERK1/2** | | | | |
| --- | --- | --- | --- | --- |
| **PI3K p110δ regulated** | **PI3K p110δ and ERK1/2 regulated** | | | **ERK1/2 regulated** |
| *Inhba* | *Timp1* | *Il1a* | *Ccl1* | *Spp1* |
| *Tnfsf14* | *Il3* | *Il6* | *Ccl9* | *Tnfsf8* |
| *Il23a* | *Csf2* | *Il31* | *Ccl22* | *Clcf1* |
| *Il22* | *Tnf* | *Cd70* |  | *Ccl4* |
| *Il21* | *Il24* | *Lta* |  | *Ccl5* |
| *Cxcl16* | *Cd40lg* | *Tnfsf9* |  | *Ccl3* |
| *Cxcl12* | *Il27* | *Il17f* |  | *Cxcl10* |
|  | *Il13* | *Il19* |  |  |
|  | *Il2* | *Il10* |  |  |
|  | *Areg* | *Xcl1* |  |  |

**References**

1. Delpoux A, Marcel N, Hess Michelini R, Katayama CD, Allison KA, Glass CK, et al. FOXO1 constrains activation and regulates senescence in CD8 T cells. Cell Rep. 2021;34(4):108674.

2. Thorvaldsdottir H, Robinson JT, Mesirov JP. Integrative Genomics Viewer (IGV): high-performance genomics data visualization and exploration. Brief Bioinform. 2013;14(2):178-92.
